# The Pathogenic Roles of Local Vitamin D Metabolism Defect in Valve Inflammation and Calcification

**DOI:** 10.1002/advs.202501250

**Published:** 2025-10-13

**Authors:** Ruichen Yang, Chong Han, Yangli Xie, Shoutao Qiu, Shaoyang Zhang, Jingjia He, Zejian Wang, Zhenlin Zhang, Huijuan Liu, Lin Chen, Baojie Li

**Affiliations:** ^1^ Bio‐X Institutes Key Laboratory for the Genetics of Developmental and Neuropsychiatric Disorders Ministry of Education Shanghai Jiao Tong University Shanghai 200240 China; ^2^ Department Of Wound Repair and Rehabilitation Medicine Center of Bone Metabolism and Repair State Key Laboratory of Trauma Burns and Combined Injury Trauma Center Research Institute of Surgery Daping Hospital Army Medical University Chongqing 400038 China; ^3^ School of Pharmacy Shanghai Jiao Tong University Shanghai 200240 China; ^4^ Metabolic Bone Disease and Genetics Research Unit Department of Osteoporosis and Bone Diseases Shanghai Jiao Tong University Affiliated Sixth People's Hospital Shanghai 200233 China; ^5^ Shanghai Institute of Stem Cell Research and Clinical Translation Shanghai 200120 China

**Keywords:** CAVD, inflammation, osteogenic, phosphate, valve, vitamin D

## Abstract

Calcific aortic valve disease (CAVD) is a highly prevalent disease that leads to heart failure. However, the pathogenesis of CAVD remains poorly understood, and the disease currently lacks medicinal treatment. In this study, utilizing a high‐phosphate‐diet‐induced valvular calcification model in conjunction with single‐cell profiling and genetic tracing, two subpopulations of *Prrx1^+^Acta2^−^* valve interstitial cells (VICs) are identified that underwent osteogenic differentiation. Mechanistically, elevated phosphate suppresses the expression of vitamin D metabolism genes primarily in VICs and response genes in immune cells, leading to local activation of CD8^+^ T cells, macrophages, and *Prox1^+^* endothelial cells in the valve. It is further shown that inflammatory cytokines and phosphate ions synergistically induced VIC osteogenic differentiation via extracellular regulated protein kinases (ERK) signaling. Administration of active vitamin D but not the inactive form suppressed inflammation and mitigated valvular calcification. Moreover, the VIC subpopulations undergoing osteogenic differentiation, suppressed expression of vitamin D metabolism and response genes, and inflammation are also observed in valve samples from patients with CAVD. This study reveals the cellular and molecular basis for valvular calcification and identifies active vitamin D as a potential drug to prevent CAVD development.

## Graphical abstract

This study identifies the valvular interstitial cell populations responsible for valvular calcification induced by hyperphosphatemia and likely aging, uncovers local vitamin D metabolism defect‐induced inflammation as a critical pathogenic factor of calcific aortic valve disease, and highlights active vitamin D and ERK inhibitor as potential preventive treatment for this disease.

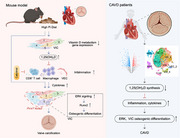

## 1 Introduction

Calcific aortic valve disease (CAVD) affects 12.6 million people globally.^[^ ^1^ ^]^ It is characterized by thickening and mineralization of the aortic valve leaflets. Ageing, high cholesterol, and dysregulated phospho‐calcium metabolism are the major risk factors.^[^ ^2^, ^3^, ^4^, ^5^, ^6^ ^]^ Currently, there is a lack of pharmacotherapy, and patients may require valve replacement.^[^ ^7^, ^8^ ^]^ CAVD is attributable to the expansion and osteogenic differentiation of valvular interstitial cells (VICs) and/or endothelial cells (VECs),^[^ ^5^ ^]^ both of which are heterogeneous.^[^ ^9^, ^10^, ^11^, ^12^, ^13^, ^14^ ^]^ Single‐cell RNA sequencing (scRNA‐seq) studies have identified several VIC subgroups in murine valves^[^ ^9^ ^]^ and distinct VIC subgroups in normal and calcified valves in humans.^[^ ^15^, ^16^, ^17^ ^]^ However, the contribution of each subgroup of VICs and VECs to CAVD development remains unclear.

The development of CAVD is closely associated with inflammation, particularly the activation of macrophages,^[^ ^18^, ^19^ ^]^ and infiltration of T cells, including CD8^+^ T cells.^[^ ^20^ ^]^ Inflammatory cytokines are thought to promote the fibrogenic and osteogenic differentiation of VICs or VECs by inducing the synthesis of transforming growth factor‐β (TGF‐β), bone morphogenetic protein (BMP), and Wnt signaling molecules.^[^ ^21^, ^22^ ^]^ Conversely, IL38, NOTCH1, and nitric oxide have been identified as inhibitors of CAVD development.^[^ ^23^, ^24^, ^25^ ^]^ Despite these insights, the precise molecular mechanisms driving valvular calcification remain incompletely understood.

Dysregulated phosphate‒calcium homeostasis is a significant risk factor for CAVD, particularly in patients with chronic kidney disease (CKD).^[^ ^26^, ^27^, ^28^, ^29^ ^]^ The prevalence of hyperphosphatemia in individuals with end‐stage renal disease (ESRD) ranges from 50% to 74%, and a study of 5036 non‐ESRD patients admitted to a tertiary care hospital found that 12% had hyperphosphatemia.^[^ ^30^, ^31^ ^]^ High phosphate (Pi) levels are a major pathogenic factor for CAVD. Pi is primarily absorbed in the small intestine, excreted by the kidneys, and reabsorbed in the renal proximal tubules. Hyperphosphatemia may promote valvular calcification through multiple mechanisms.^[^ ^32^ ^]^ Pi can form calcium‐phosphate crystals and generate reactive oxygen species (ROS) that damage valvular cells, thereby initiating inflammation. Both inflammatory cytokines and ROS stimulate the osteogenic differentiation of valvular cells.^[^ ^33^ ^]^ Alternatively, Pi may bind to phosphate transporters such as Pit1, Pit2, and NaPiT, activating nuclear factor kappa‐light‐chain‐enhancer of activated B cells (NF‐κB) signaling in valvular cells. This enhances BMP expression and promotes osteogenic differentiation, contributing to CAVD pathogenesis.^[^ ^18^ ^]^

Vitamin D is a key regulator of Pi homeostasis.^[^ ^26^, ^34^ ^]^ It is obtained from sun exposure and diet, metabolized to 25‐hydroxyvitamin D (25(OH)D) in the liver by CYP2R1 and CYP27A1, and then converted to its active form, 1,25‐dihydroxyvitamin D (1,25(OH)_2_D), in the kidney by CYP27B1. In some CKD patients, serum vitamin D levels are reduced, which has been associated with inflammation.^[^ ^35^ ^]^ However, recent studies showed that supplementation with vitamin D or 1,25(OH)_2_D shows limited benefit in improving outcomes.^[^ ^36^, ^37^, ^38^ ^]^ Therefore, the exact role of vitamin D in the pathogenesis of CAVD requires further study.

To better understand the pathogenesis of CAVD, particularly in the context of high Pi levels, we used a combination of mouse models, lineage tracing, scRNA‐seq, VIC cultures, and transcriptome analysis of CAVD patient samples. We found that high Pi disrupts the expression of vitamin D metabolism genes, leading to lowered levels of 1,25(OH)_2_D. This promotes the expansion of CD8^+^ T cells and macrophages, causing inflammation in the valve. Inflammatory cytokines together with Pi synergistically induced osteogenic differentiation of two *Prrx1^+^Acta2^−^* VIC subpopulations via extracellular regulated protein kinases (ERK) signaling. This process was reduced by treatment with active vitamin D, but not with inactive vitamin D, or by using an ERK inhibitor. Importantly, vitamin D metabolism defects, inflammation, ERK and other signaling pathways, and the specific VIC subgroups undergoing calcification were observed in patients with CAVD of unknown cause, aside from advanced age. Since aging is a major risk factor for both CAVD and vitamin D metabolism impairment, our findings suggest that vitamin D metabolism defects may be a common pathogenic factor in CAVD. Therefore, active vitamin D may have potential as a preventative treatment for this condition.

## 2 Results

### 2.1 High‐Phosphate‐Diet (HPD) ‐Induced Valvular Calcification Is Associated with Osteogenic Differentiation and Inflammation

**Figure 1:**
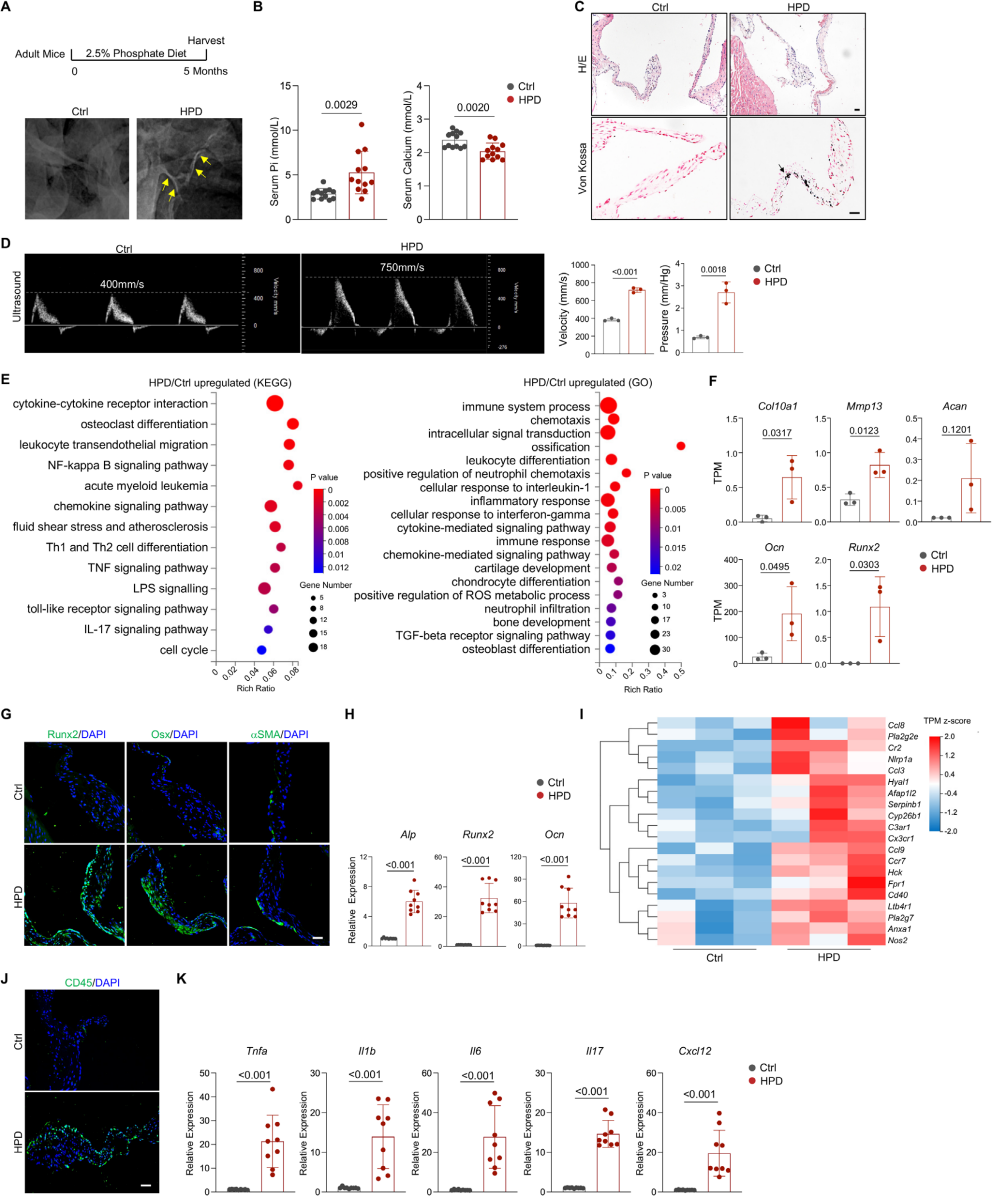
HPD‐induced valvular calcification was associated with inflammation. A) X‐ray imaging revealed that HPD caused calcification of the aortic valves in adult male mice. Upper panel: Schedule for HPD treatment. B) HPD led to an increase in the serum phosphate levels and a modest decrease in serum calcium levels. N = 6 mice, with duplicates each. C) Representative H/E and Von Kossa staining results showing disrupted structure and mineralization nodules in the valve of HPD mice. Scale bars = 25 µm. D) The HPD mice displayed increased transaortic valve pressure and transaortic valve velocity, detected by ultrasound. N = 3. E) KEGG and GO analysis results showing enriched pathways or modules in the valve cells of HPD mice. N = 3. F) TPM values of osteogenic or chondrogenic genes expressed in valve samples of HPD and control mice. N = 3. G) Representative immunostaining results for Runx2, Osx, and αSMA in the valve of HPD and control mice. Scale bars = 25 µm. H) qPCR analysis of osteoblast related genes in the valve samples of HPD and control mice. N = 3 mice, with triplicate each. I) Heatmaps of major inflammation‐related genes. J) Representative staining results for CD45^+^ immune cells. Scale bars = 25 µm. K) qPCR analysis of cytokines and chemokines expressed in the valve samples of HPD and control mice. N = 3 mice, with triplicate each. An unpaired two‐tailed Student's *t*‐test was applied to evaluate the correlation data in (B, D, F, H, and K), *p* < 0.05 was considered statistically significant.

To understand the pathogenesis of CAVD, we utilized hyperphosphatemia as an inducer of valve calcification. We fed adult male mice chow containing 2.5 or 5% phosphate or calcium over a period of 2–5 months, as previously described.^[^ ^18^, ^39^ ^]^ We found that HPD (2.5% or 5%), but not a high‐calcium diet, caused calcification in aortic valves after 4–5 months on HPD and was detectable by X‐ray imaging (**Figure** 1A; Figure S1A,B, Supporting Information). At three months on HPD, calcification was only detectable by histological analysis, not by X‐ray imaging. HPD led to an 82.8% increase in the serum phosphate level and a 14.2% decrease in calcium level (Figure 1B). Histological analysis revealed disrupted valve architecture, and Von Kossa staining revealed mineralization nodules, especially on the aortic side of the valves (Figure 1C). Functionally, HPD led to elevated transaortic valve pressure and transaortic valve velocity, as detected by ultrasound imaging (Figure 1D).

To elucidate the molecular mechanisms underlying these valvular changes, we performed bulk RNA sequencing on valve samples from HPD and control mice (Figure 1E). Kyoto encyclopedia of genes and genomes (KEGG) and Gene ontology (GO) analyses revealed enriched pathways or modules, including skeletal development, chondrocyte differentiation, cartilage development, ossification, BMP signaling, toll‐like receptor (TLR), NF‐κB, IL17, tumor necrosis factor (TNF), monocyte and T‐cell chemotaxis, LPS signaling, T cell proliferation, and neutrophil infiltration, in HPD mice (Figure 1E; Figure S2A, Supporting Information).

The enriched genes associated with cartilage development and ossification included *Col10*, *Acan* (Aggrecan), *Mmp13*, *Runx2*, and *Ocn* (Osteocalcin) (Figure 1F). Immunostaining revealed increased expression of osteoblast‐specific Runx2 and Osx but not the myofibroblast‐specific αSMA in the valves of HPD mice (Figure 1G). qPCR results confirmed that the expression of osteoblast markers such as *Alp*, *Runx2*, and *Ocn*, and some *BMPs* and *Wnts* was elevated in the valve of HPD mice (Figure 1H; Figure S2B, Supporting Information).

We also observed increased expression of inflammatory cytokines and chemokines in the valves of HPD mice (Figure 1I; Figure S2A, Supporting Information). Immunostaining revealed infiltration of CD45^+^ immune cells before or after calcification occurred (Figure 1J; Figure S2C, Supporting Information). We also detected increases in the expression of *TNFα*, *Il1b*, *Il6*, *Il17*, and *Cxcl12* via qPCR and in the levels of phosphorylated RelA (an NF‐κB subunit) and Stat3, two major signaling molecules downstream of various cytokines or chemokines, via immunostaining (Figure 1K; Figure S2D, Supporting Information).

Overall, these findings indicated that high phosphate levels induced valvular calcification, which is associated with osteogenic and chondrogenic differentiation of valvular cells, as well as inflammation.

### 2.2 HPD Induces Osteogenic Differentiation of Two Subgroups of VICs

**Figure 2:**
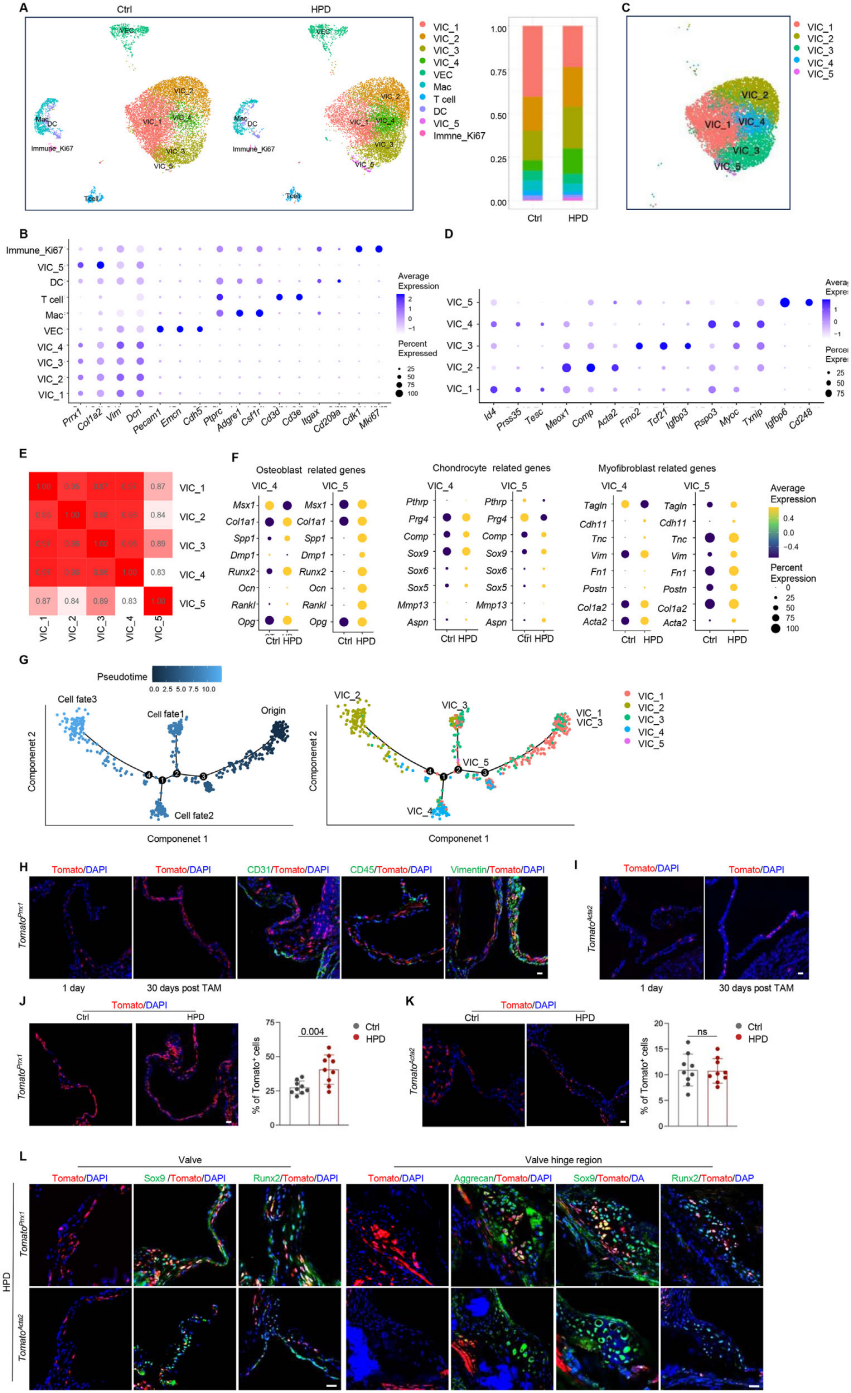
scRNA‐seq and genetic tracing analyses of valvular cells from HPD and control mice. A) t‐SNE analysis of valvular cells from HPD and control mice. Valve cells from 10 mice were combined for 10x Genomics scRNA‐seq. Right panel: the proportions of various cell populations in HPD and control mice. B) Markers used for clustering of valvular cell types. C) t‐SNE analysis of VICs. D) Markers used for clustering of VICs. E) Heatmap of the top 200 genes expressed in various VIC groups. R^2^ indicates the similarity among different VIC clusters. F) Expression of major osteoblast, chondrocyte, and myofibroblast signature genes in VIC groups 4 and 5. G) Pseudotime trajectory analysis of the 5 VIC groups. H) Genetic tracing of valvular cells in *Tomato^Prrx1^* mice. Adult male mice were euthanized on Day 1 or 30 after TAM administration. The valve sections were coimmunostained with Tomato, DAPI, and antibodies against CD31, CD45, or Vimentin. Scale bars = 25 µm. I) Genetic tracing of valvular cells in *Tomato^Acta2^* mice, as described in (H). Scale bars = 25 µm. J) Immunostaining revealed an increase in *Prrx1^+^* lineage cells in adult male *Tomato^Prrx1^* mice subjected to HPD for 4 months. Right panel: quantitation of Tomato^+^ VICs in HPD and control mice. N = 3. Scale bars = 25 µm. K) Immunostaining revealed no alteration in *Acta2^+^* lineage cells in adult male *Tomato^Acta2^* mice subjected to HPD for 4 months. Right panel: quantitation of Tomato^+^ VICs in HPD and control mice. N = 3. Scale bars = 25 µm. L) Representative costaining results revealed that Tomato^+^ cells expressed Runx2, Aggrecan, or Sox9 in *Tomato^Prrx1^* but not in *Tomato^Acta2^* mice. Scale bars = 25 µm. An unpaired two‐tailed Student's *t*‐test was applied to evaluate the correlation data in (J and K), *p* < 0.05 was considered statistically significant.

We isolated valvular cells from 10 HPD or control mice and conducted single‐cell RNA sequencing (scRNA‐seq). t‐SNE analysis revealed 5 VIC groups, a macrophage group, a T cell group, a dendritic cell group, a VEC group, and a proliferative immune cell group (**Figure** 2A,B). The proportions of some cell populations were altered by HPD (Figure 2A); however, no novel cell population emerged.

We reanalyzed the VICs in detail using the markers described in previous studies^[^ ^40^ ^]^ and detected an expansion of VIC groups 4 and 5 (Figure 2C,D). Analysis of the top 200 genes expressed revealed that the 5 groups presented very similar gene expression profiles (Figure 2E), suggesting that they might have the same identity. We compared each of the stromal subgroups of HPD mice to those of control mice and found that all 5 subgroups expressed some osteoblast‐, chondrocyte‐, ossification‐, and skeletal development‐related genes in response to HPD (Figure S3A, Supporting Information), suggesting that they might have initiated osteogenic differentiation to various degrees. A comparison of the expression of the major osteoblast, chondrocyte, and myofibroblast signature genes revealed that subgroups 4 and 5 displayed the highest levels of osteogenic and chondrogenic gene expression (Figure 2F; Figure S3B, Supporting Information). These results, in conjunction with the observation that the VICs of subgroups 4 and 5 exhibited expansion in HPD mice, indicated that these two subgroups of VICs were primarily responsible for valvular calcification under hyperphosphatemia conditions. Pseudotime trajectory analysis revealed that the group 4 and 5 groups of VICs are likely derived from the 1 and 3 groups of VICs (Figure 2G).

### 2.3 Genetic Tracing Reveals That Prrx1^+^Acta2^−^ VICs Are Involved in Calcification

VICs are hypothesized to be of mesenchymal/stromal origin. To investigate this possibility, we examined the expression of common mesenchymal stromal/stem or skeletal stem cell markers using the scRNA‐seq data. Our analysis revealed that *Acta2* and *Nestin* were predominantly expressed in group 2, whereas *Prrx1*, *Pdgfra*, *Col1a2*, and *Vimentin* were expressed across nearly all subgroups, to various degrees. Conversely, *Lepr* and *Gli1* were detected in small portions of valvular cells (Figure 2C; Figure S4A, Supporting Information). We subsequently performed genetic lineage tracing of valvular cells in adult *Tomato^Prrx1^* mice and *Tomato^Acta2^* mice (Figure 2H,I).^[^ ^41^, ^42^ ^]^ The results revealed that *Prrx1* marked the majority of VICs, but not VECs, 30 days after tamoxifen (TAM) injection in adult mice (Figure 2H), as the Tomato^+^ cells were CD31^−^ and CD45^−^, but Vimentin^+^ (Figure 2H). *Prrx1* may be a genetic marker for all VICs, consistent with the observation that the 5 VIC subgroups present similar gene expression profiles (Figure 2E). In addition, our genetic tracing experiments showed that *Vimentin* marked all valvular cells, whereas *Col1a2* or *Gli1* marked only a subset of valvular cells (Figure S4B–D, Supporting Information). These *CreERT^2^* mouse lines may be useful in studying the functions of the heterogeneous VICs.

We then fed *Tomato^Prrx1^* and *Tomato^Acta2^* mice HPD for 4 months, and we observed an expansion of *Prrx1^+^* lineage cells (Tomato^+^) but not *Acta2^+^* lineage cells in the valve (Figure 2J,K). Furthermore, costaining revealed that the Tomato signal overlapped with that of Runx2 or chondrocyte‐specific Acan in the valves of *Tomato^Prrx1^* mice but not *Tomato^Acta2^* mice (Figure 2L). Overall, these results suggested that *Prrx1^+^Acta2^−^* VICs were involved in valvular calcification in response to HPD, which was consistent with the scRNA‐seq results showing that *Acta2* was not expressed in subgroup 4 or 5 VICs (Figures 2C,D), the major VIC subgroups contributing to valvular calcification.

### 2.4 Enrichment of CD8^+^ T Cells in the Valves but not the Peripheral Blood of HPD Mice

**Figure 3:**
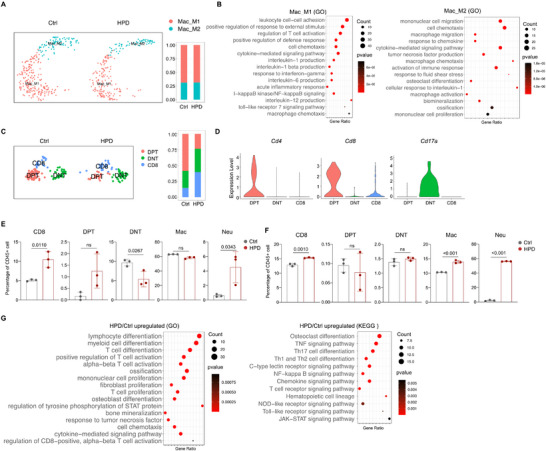
HPD mice presented increased CD8^+^ T cells in the valve but not the peripheral blood. A) scRNA‐seq analysis of macrophages in the valves of HPD mice. B) GO analysis of macrophage subgroup 1 and subgroup 2. C) scRNA‐seq analysis revealed an increase in CD8^+^ T cells. D) Markers used to classify T cell subgroups, with *Cd17a* being used as a DNT marker. E) Flow cytometry results of the percentages of DNT, DPT, CD8^+^ T cells, macrophages, and neutrophils in the valve of HPD and control mice. N = 3. F) Flow cytometry results of the percentages of DNT, DPT, CD8^+^ T cells, macrophages, and neutrophils in the peripheral blood of HPD and control mice. N = 3. G) KEGG and GO analysis of CD4^−^CD8^+^ T cells from HPD mice compared to control mice. An unpaired two‐tailed Student's *t*‐test was applied to evaluate the correlation data in (E and F), *p* < 0.05 was considered statistically significant.

Further scRNA‐seq analysis revealed that macrophages could be divided into 2 subgroups (**Figure** 3A). Subgroup 2 macrophages exhibited gene expression features suggestive of osteoclastogenesis and pro‐osteogenic activities (Figure 3B). Both macrophage groups were activated by HPD and exhibited increased expression of inflammatory cytokines (Figure S5A,B, Supporting Information). Moreover, valvular T cells could be divided into double negative T (DNT), double positive T (DPT), and CD4^−^CD8^+^ T subgroups (Figure 3C,D). HPD led to a reduction of DPT cells and an increase CD4^−^CD8^+^ T cells (Figure 3C,D). CD8^+^ T cells have been reported in the calcified valves of patients with CAVD, although their functions remain unclear.^[^ ^20^ ^]^

We then analyzed the immune cells of the valves of HPD and control mice by flow cytometry and found that the number of CD4^−^CD8^+^ T cells and DPT cells increased, DNT cells decreased, while macrophages showed no significant change (Figure 3E; Figure S5C,D, Supporting Information). Although scRNA‐seq did not detect neutrophils, flow cytometry analysis revealed an increase in neutrophils in the valve of HPD mice (Figure 3E). The discrepancies between scRNA‐seq and flow cytometry results may be caused by insufficient depth of sequencing. For comparison, we analyzed immune cells in the peripheral blood and detected an increase in the number of neutrophils, macrophages, and CD4^−^CD8^+^ T cells, but not DPT cells or DNT cells, in HPD mice compared to control mice (Figure 3F; Figure S5C,E, Supporting Information). These differences in the valve and peripheral blood suggested that HPD might affect valve‐resident CD4^−^CD8^+^ T cells in the valve locally.^[^ ^20^ ^]^ Although CD8^+^ T cells are mainly in charge of immune surveillance, resident CD4^−^CD8^+^ T cells have been reported to secrete proinflammatory chemokines and cytokines.^[^ ^43^, ^44^, ^45^ ^]^ Our scRNA‐seq analysis revealed that CD4^−^CD8^+^ T cells exhibited enriched expression of genes in lymphocyte inflammation, myeloid differentiation, T cell proliferation and differentiation, TNF signaling, NF‐κB signaling, TLR signaling, Jak‐Stat signaling in the valve of HPD mice (Figure 3G), confirming their pro‐inflammation activities.

scRNA‐seq analysis of endothelial cells revealed three distinct subpopulations (Figure S6A,B, Supporting Information), as identified through KEGG and GO pathway enrichment. Although scRNA‐seq data indicated a modest increase in *Prox1^+^* endothelial cells, immunostaining for lymphatic endothelial cells showed no noticeable change (Figure S6A,C, Supporting Information). GO analysis revealed that *Prox1^+^* endothelial cells expressed high levels of cytokines and chemokines compared to the other two subgroups (Figure S6D,E, Supporting Information). These results align with the well‐established role of Prox1 in promoting inflammation within lymphatic endothelial cells.^[^ ^46^, ^47^ ^]^ Overall, these findings suggested a combined contribution of immune cells and endothelial cells to the inflammatory processes observed in the valves of HPD mice. However, the role of lymphatic endothelial cells in inflammation requires further investigation.

### 2.5 HPD Suppressed the Expression of Vitamin D Metabolism Genes Primarily in VICs

**Figure 4:**
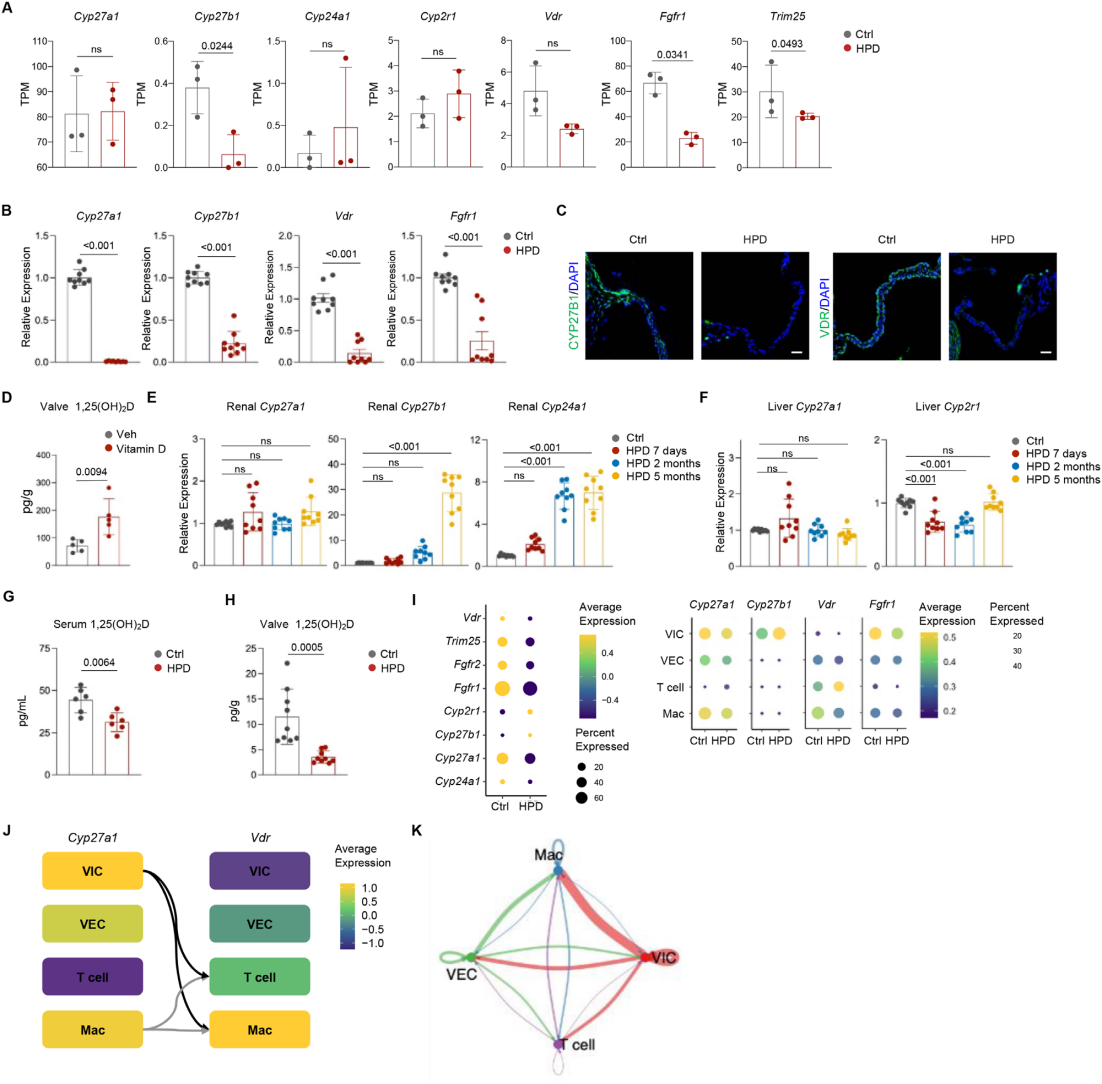
HPD mice presented reduced expression of vitamin D metabolism and response genes in the valve. A) Bulk RNA‐seq revealed a decrease in the expression of a number of vitamin D metabolism and response genes in the valves of HPD mice. TPM values of the related genes were presented. N = 3. B) qPCR analysis confirmed a decrease in the expression of a number of genes involved in vitamin D metabolism and response genes. N = 3 mice, with triplicate each. C) Representative immunostaining results for CYP27B1 and VDR in the valve of HPD and control mice. Scale bars = 25 µm. D) ELISA assays showing that Vitamin D is metabolized into its active form, 1,25(OH)_2_D, in ex vivo valve cultures. N = 5. E) qPCR analysis of vitamin D metabolism genes in kidney samples from HPD and control mice. N = 3 mice, with triplicate each. F) qPCR analysis of vitamin D metabolism genes in the liver samples from HPD and control mice. N = 3 mice, with triplicate each. G) ELISA assays showed that HPD led to a decrease in the serum levels of 1,25(OH)_2_D. N = 6. H) ELISA assays revealed a great decrease in active vitamin D in valve samples from HPD mice. N = 9. I) scRNA‐seq analysis revealed changes in the expression of vitamin D metabolism and response genes in combined valvular cells or various cell populations. J) *Cyp27a1‐Vdr* interaction analysis in valvular cell populations. K) Overall ligand‐receptor interaction analysis revealed communications among all cell groups with VICs as the major signal‐sending cells. The thickness of the lines indicates the greatness of the interaction. The color of the lines indicates the signals sent from the cells with the same color. An unpaired two‐tailed Student's *t*‐test was applied to evaluate the correlation data in (A, B, D‐H), *p* < 0.05 was considered statistically significant.

To investigate the role of inflammation in HPD‐induced valvular calcification, we initially administered dexamethasone to the mice. However, concurrent administration of dexamethasone and HPD resulted in significant debilitation of the mice, leading to mortality within one month (data not shown). We also tested LDN, a BMPR1A inhibitor, since BMP signaling plays a key role in osteoblast differentiation. However, LDN did not affect valvular calcification induced by HPD. Bulk RNA sequencing revealed a reduction in some genes associated with vitamin D metabolism and response (**Figure** **4**A). Our qPCR assays verified that only *Cyp27a1*, *Cyp27b1*, *Vdr*, and *Fgfr1* were significantly downregulated in the valves of HPD mice (Figure 4B). Immunostaining confirmed reduced protein levels of CYP27B1 and VDR in the valves of mice treated with HPD (Figure 4C). Additionally, we demonstrated that ex vivo aortic valve cultures could synthesize active vitamin D (1,25(OH)_2_D) from vitamin D (Figure 4D). These findings are consistent with reports that vitamin D metabolic enzymes are expressed in tissues other than the liver and kidney, where 1,25(OH)_2_D is synthesized to execute autocrine or paracrine functions.^[^ ^44^, ^48^ ^]^

Conversely, the expression of *Cyp27b1* and *Cyp24a1* increased while the expression of *Cyp27a1* did not significantly change in the kidney of HPD mice (Figure 4E). In the liver, *Cyp2r1* expression slightly decreased while *Cyp27a1* did not significantly change in HPD mice (Figure 4F). It has been reported that the expression of vitamin D metabolism genes in the kidney is elevated in response to high phosphate.^[^ ^39^ ^]^ Expression of *Cyp27a1* in the kidney may serve as a compensatory mechanism when vitamin D metabolism is impaired in the liver.

Hyperphosphatemia is known to cause a decrease in serum vitamin D.^[^ ^35^, ^49^ ^]^ We found that HPD mice displayed 70.6% of the normal serum levels of 1,25(OH)_2_D (Figure 4G). We also analyzed the valve samples for 1,25(OH)_2_D and found that HPD mice displayed 30.5% of normal 1,25(OH)_2_D levels (Figure 4H). Collectively, these findings suggested that HPD impaired the synthesis and response of active vitamin D in the valve, which is likely to act on immune cells or VICs in autocrine and paracrine manners. The systemic reduction of active vitamin D, although less severe than in the valve, may also contribute to valvular calcification.

Analysis of the scRNA‐seq data of total valvular cells combined also revealed suppressed expression of certain vitamin D metabolism and response genes in HPD mice compared to control mice (Figure 4I). Further analysis revealed that the expression of *Cyp27a1* was reduced primarily in VICs while the expression of *Vdr* was reduced primarily in macrophages (Figure 4I). *Cyp27a1‐Vdr* interaction analysis confirmed a communication from VICs to macrophages and T cells (Figure 4J). Overall ligand‐receptor interaction analysis revealed communications among all cell groups, with VICs being the major signal‐sending cells (Figure 4K). Moreover, we found that inflammatory signals from macrophages or T cells affected VICs, while the signals from CD8^+^ T cells influenced other T cells (Figure S7A–C, Supporting Information). These results suggest that VIC‐synthesized active vitamin D may act on macrophages and T cells to suppress inflammation, and that inflammation‐related molecules secreted by immune cells may act on VICs to induce osteogenic differentiation.

### 2.6 Active Vitamin D Prevents Valvular Calcification and Inflammation

**Figure 5:**
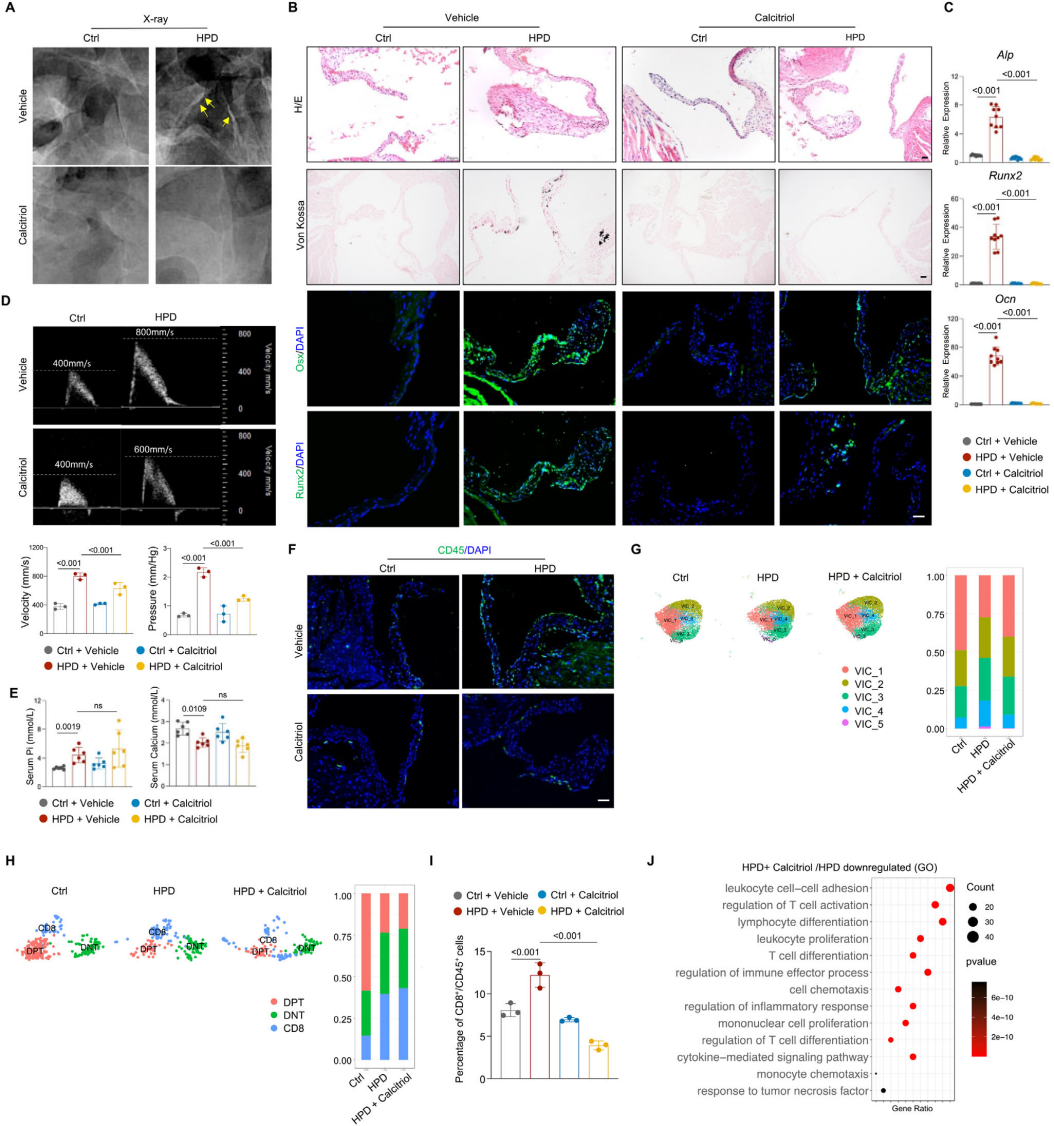
Active vitamin D mitigated valve inflammation and calcification. A) X‐ray images showing valval calcification in the Ctrl + Vehicle, HPD + Vehicle, Ctrl + Calcitriol, and HPD + Calcitriol mouse groups. B) H/E and Von Kossa staining and immunostaining for Runx2 and Osx in the valves from the four groups of mice. Scale bars = 25 µm. C) qPCR results showed that calcitriol suppressed the expression of osteogenic genes in the valves of HPD mice. N = 3 mice, with triplicate each. D) HPD‐induced increases in transaortic valve pressure and transaortic valve velocity were mitigated by calcitriol. N = 3. E) The effects of calcitriol on the serum Pi or calcium levels in HPD and control mice. N = 6. F) Calcitriol blocked CD45^+^ immune cell infiltration into the valve in HPD mice. Scale bars = 25 µm. G) t‐SNE analysis of VICs in calcitriol‐treated mice on HPD. Right panel: the percentages of various cell subgroups. H) t‐SNE analysis of T cells in calcitriol‐treated mice on HPD. Right panel: the percentages of various cell subgroups. I) Flow cytometry results showed that calcitriol suppressed the increase in CD8^+^ T cells in the valve of HPD mice. N = 3. The experiments were done as described in Figure S5C,D (Supporting Information). J) GO analysis results showed that the expression of inflammatory cytokine in CD8^+^ T cells was suppressed by calcitriol. Two‐way ANOVA (or mixed model) multiple comparisons were applied to evaluate the correlation data in (C, D, E, and I), *p* < 0.05 was considered statistically significant.

To test the significance of 1,25(OH)_2_D metabolism defect in HPD‐induced valvular calcification, we treated HPD mice with calcitriol, an active form of vitamin D, or vitamin D, for 4 months (See methods for dose selection). We found that a dose of 100 ng kg^−1^ nearly completely prevented valve calcification in HPD mice. Lower doses were also tested: 20 ng kg^−1^ had no effect, while 50 ng kg^−1^ offered partial protection (**Figure** 5A; Figure S8A, Supporting Information). Histological analysis confirmed that valvular calcification was suppressed by 100 ng kg^−1^ calcitriol, accompanied by decreases in the expression of Runx2 and Osx (Figure 5B). qPCR revealed suppressed expression of *Alp*, *Runx2*, and *Ocn* in the valve of HPD mice (Figure 5C). Moreover, the HPD‐induced increase in transaortic valve pressure and transaortic valve velocity was mitigated (Figure 5D). However, inactive vitamin D had no effect on valvular calcification (Figure S8B,C, Supporting Information), which was consistent with the results of clinical trials showing no beneficial effect of vitamin D in cardiovascular patients.^[^ ^37^, ^50^ ^]^ We also measured serum levels of vitamin D and 25(OH)D, finding that HPD increased both isoforms (Figure S8D, Supporting Information). These results further support that vitamin D metabolism is disrupted in HPD mice. Overall, these findings implied that a shortage of active vitamin D, especially within valves, might play a causal role in valvular calcification in HPD mice.

Vitamin D is known to increase serum Pi levels; we found that calcitriol induced an insignificant increase in serum Pi levels and an insignificant decrease in serum calcium levels in HPD or control mice (Figure 5E). Immunostaining further revealed that calcitriol inhibited CD45^+^ immune cell infiltration in valve sections (Figure 5F). These results suggest that calcitriol suppressed inflammation. In addition, qPCR analysis revealed that the increased expression of *Wnt* or *BMP* molecules in the valves of HPD mice was suppressed by calcitriol (Figure S8E, Supporting Information).

We subsequently conducted a scRNA‐seq analysis of the valvular cells in HPD mice treated with calcitriol (Figure S8F, Supporting Information). Detailed analysis showed that VIC subgroup 5, which presented the highest osteogenic potential, was diminished following calcitriol treatment (Figure 5G). Although we did not observe a reduction of CD4^−^CD8^+^ T cells in scRNA‐seq data, our flow cytometry analysis showed that the percentage of CD4^−^CD8^+^ T cells was reduced by calcitriol (Figure 5H,I). Moreover, scRNA‐seq analysis revealed that calcitriol suppressed the activation of CD4^−^CD8^+^ T cells in HPD mice (Figure 5J). In addition, calcitriol also mitigated the enhanced expression of inflammatory cytokines in endothelial cells in HPD mice (Figure S8G–I, Supporting Information). Overall, these results indicated that calcitriol prevented changes in VICs, VECs, and immune cells associated with valvular calcification.

### 2.7 Inflammation and Pi Synergistically Induce VIC Osteogenic Differentiation

**Figure 6:**
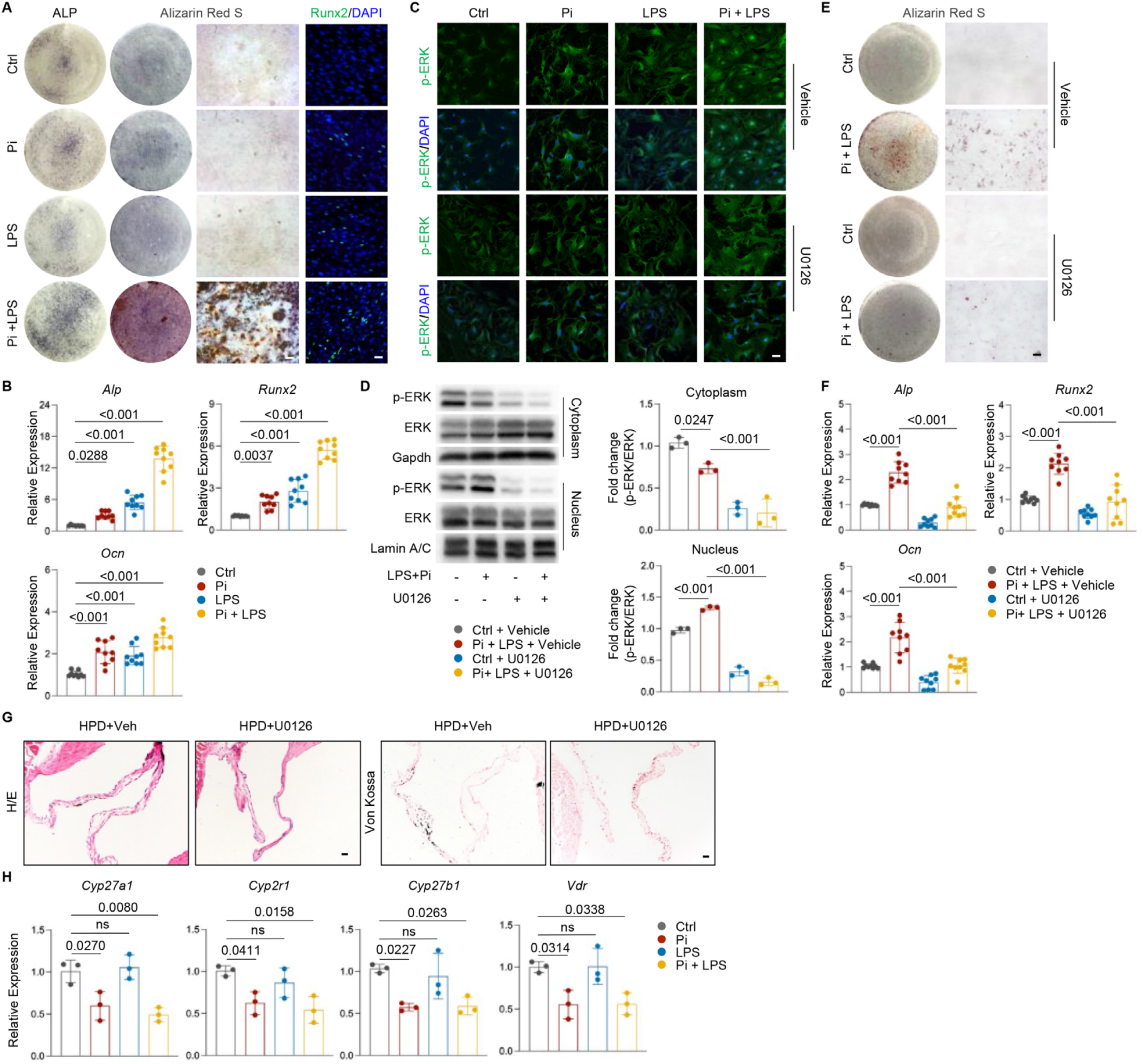
Pi and inflammation cooperated to induce VIC osteogenic differentiation. A,B) Pi and LPS showed additive effects on osteogenic differentiation of VICs, as verified by ALP, Alizarin red staining or immunostaining of Runx2 (A) and qPCR analysis of osteoblast‐specific markers (B). N = 3, with triplicate each. Scale bars = 100 µm. C) Pi and LPS induced nuclear localization of activated ERKs in VICs, which was suppressed by U0126. Scale bars = 25 µm. D) Treatment with Pi (4 mm) and LPS induced the nuclear entry of activated ERKs and caused a slight decrease in cytoplasmic levels in VICs. In contrast, U0126 effectively inhibited p‐ERK signaling in both the cytoplasm and nucleus of VICs. Cells were collected 6 h after treatment. Right panel: quantitation data. N = 3. E,F) U0126 prevented Pi and LPS‐induced osteogenic differentiation of VICs, as confirmed by Alizarin red staining (E) and qPCR analysis of osteoblast‐specific markers (F). N = 3, with triplicate each. Scale bars = 100 µm. G) Representative H/E and Von Kossa staining results showed that HPD‐induced valvular calcification could be largely prevented by U0126. Scale bars = 25 µm. H) qPCR analysis of active vitamin D synthesis genes in primary VIC cultures treated with Pi, LPS or both. N = 3. Two‐way ANOVA (or mixed model) multiple comparisons were applied to evaluate the correlation data in (B and E), an unpaired two‐tailed Student's *t‐test* was applied to evaluate the correlation data in (F), *p* < 0.05 was considered statistically significant.

The following question arises: how does HPD induce VIC osteogenic differentiation? Previous studies have shown that Pi can directly induce the osteogenic differentiation of vascular smooth muscle cells.^[^ ^51^ ^]^ We tested cultured primary mouse valve cells, which showed minimal osteogenic differentiation, and found that Pi promoted VIC osteogenic differentiation, as confirmed by histological staining and qPCR results for osteoblast‐specific markers such as *Alp*, *Runx2*, and *Ocn* (Figure S9A,B, Supporting Information). We also found that LPS (a pro‐inflammatory agent) stimulated osteogenic differentiation of VICs, to a lesser extent than Pi (Figure S9C,D, Supporting Information). Moreover, low doses of Pi and LPS showed additive effects on VIC osteogenic differentiation (**Figure** 6A,B). Immunostaining showed that Runx2 expression was increased following treatment with Pi and LPS (Figure 6A). These results suggested that Pi ions and inflammatory cytokines might synergistically induce VIC osteogenic differentiation and subsequent valvular calcification.

Our transcriptome analysis of VICs (combining all five subsets) revealed that HPD activated ERK, Wnt, TGF‐β, Akt‐mTOR, and BMP signaling pathways, along with inflammation (Figure S10A, Supporting Information). However, treatment of HPD mice with the BMPR1A antagonist LDN did not prevent valvular calcification. Previous studies have shown that Pi induces the osteogenic differentiation of vascular smooth muscle cells via NF‐κB signaling.^[^ ^51^ ^]^ However, Pi did not activate NF‐κB in VIC cultures, and inhibition of NF‐κB with QNZ did not affect Pi‐induced VIC osteogenic differentiation (Figure S10B–D, Supporting Information). Furthermore, we found that blocking β‐catenin activation with IWR‐1 did not affect phosphate and LPS‐induced osteogenic differentiation of VICs (Figure S10E,F, Supporting Information). It is likely that these pathways have redundant functions in promoting VIC osteogenic differentiation.

Additionally, Pi and LPS resulted in increased nuclear localization of p‐ERK, although Western blot revealed no significant alteration of total p‐ERKs levels in VIC cultures (Figure 6C,D; Figure S10G,H, Supporting Information). Moreover, blockade of ERK with U0126 suppressed Pi and LPS‐induced VIC osteogenic differentiation (Figure 6E,F). We also treated HPD mice with U0126 and found that it largely prevented valvular calcification (Figure 6G). These results highlight the importance of ERK signaling in valvular calcification, especially by the nuclear targets of ERKs.^[^ ^52^ ^]^

We also investigated the possible direct impact of calcitriol on VIC osteogenic differentiation. We treated VICs with calcitriol, and our staining and qPCR results revealed that calcitriol did not significantly affect Pi and LPS‐induced VIC osteogenic differentiation (Figure S10I,J, Supporting Information). High doses of calcitriol did not affect Pi and LPS‐induced expression of *Runx2* (Figure S10K, Supporting Information). These results suggest that calcitriol does not directly affect VIC differentiation; rather, it inhibits inflammation and cytokine expression, which in turn promotes VIC osteogenic differentiation.

Moreover, we found that high phosphate, but not LPS, suppressed the expression of *Cyp27a1* and *Cyp2r1*, encoding enzymes for 25(OH)D synthesis from vitamin D, and *Cyp27b1*, encoding enzymes for 1,25(OH)_2_D synthesis from 25(OH)D, in primary VICs (Figure 6H), supporting that Pi directly suppresses the expression of vitamin D metabolism genes, consistent with our RNA‐seq and qPCR data from valve samples.

### 2.8 CAVD Patient Samples Present Vitamin D Metabolism Defects and Inflammation

**Figure 7:**
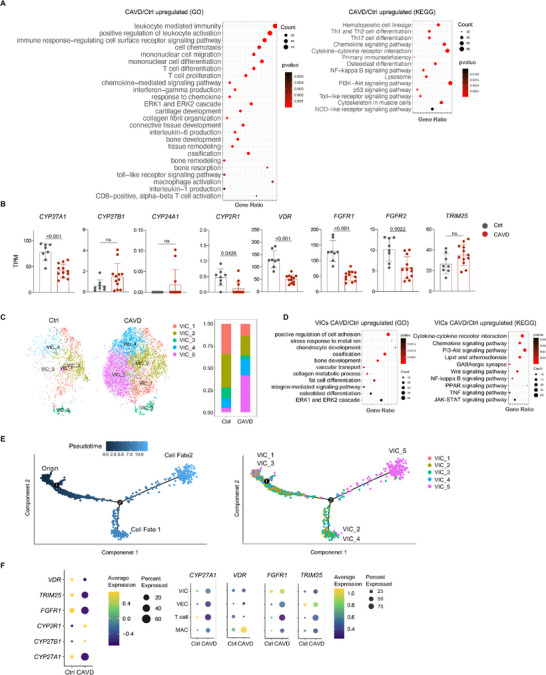
Human CAVD samples presented defects in vitamin D metabolism and response. A) KEGG and GO analyses of bulk RNA‐seq data (GSE148219) revealed increased expression of genes related to cell proliferation, osteogenic and chondrogenic differentiation, ossification, and inflammation in valve samples from patients with CAVD. N = 8 for control and N = 12 for patients. B) The expression of vitamin D metabolism and response genes in the valve samples of patients with CAVD. N = 8 for control and N = 12 for patients. C) t‐SNE analysis of scRNA‐seq data from patients with CAVD (PRJNA562645). The right panel: the percentages of various VIC groups. D) KEGG and GO analyses of VICs (combined) from scRNA‐seq data of patients with CAVD and healthy individuals (PRJNA562645). N = 2 for control and N = 4 for patients. E) Pseudotime trajectory analysis of the 5 VIC groups of humans. F) scRNA‐seq analysis of some vitamin D metabolism and response genes in the valve samples of patients and normal controls. The valvular cells were combined for the patients or the control. An unpaired two‐tailed Student's *t*‐test was applied to evaluate the correlation data in (B), *p* < 0.05 was considered statistically significant.

Finally, we tested whether vitamin D metabolism and response defects in the valve were present in patients with CAVD. We analyzed the bulk RNA‐seq datasets deposited in the public domain (12 patients and 8 controls).^[^ ^53^ ^]^ Although the pathogenesis of these patients was unknown, a link between chronic inflammation and biological ageing was suggested.^[^ ^53^ ^]^ Our KEGG and GO analyses confirmed increased expression of genes related to cell proliferation, osteogenic and chondrogenic differentiation, ossification, and inflammation (**Figure** **7A**). In addition, the expression of vitamin D metabolism genes, such as *CYP27A1* and *CYP2R1*, was downregulated in the valve samples of patients with CAVD, as was the expression of vitamin D response genes, including *VDR*, *FGFR1*, and *FGFR2* (Figure 7B). We analyzed another bulk RNA‐seq dataset from CAVD patients and controls (average age 60.4 years).^[^ ^54^ ^]^ The analysis revealed similar changes in the expression of genes related to skeletal development and inflammation (Figure S11A, Supporting Information). Additionally, some vitamin D metabolism and response genes were downregulated in these patient samples (Figure S11B, Supporting Information). Overall, these results suggest that both mice and humans with valve calcification have reduced synthesis of active vitamin D in the valve. Since vitamin D metabolism deficiency is common in the elderly,^[^ ^55^, ^56^ ^]^ vitamin D metabolism and response defects may be a common pathogenic factor in hyperphosphatemia‐ and ageing‐induced valvular calcification.

We then reanalyzed the deposited scRNA‐seq datasets of valve samples from patients with CAVD (Figure S12A,B, Supporting Information).^[^ ^15^, ^16^ ^]^ We found that the VICs could be divided into 5 subgroups, with subgroups 4 and 5 showing expansion (Figure 7C), similar to the VIC groups 4 and 5 in the mice. GO analysis revealed that group 1 and 3 VICs showed enriched expression of genes specific to multiple lineages, including osteoblast, chondrocyte, adipocyte, and mesenchymal cell (Figure S12C, Supporting Information), suggesting that they might be multipotential. Analysis of all VICs combined revealed that patient samples exhibited enriched gene expression related to ERK, mTOR, Wnt, and NF‐κB pathways, as well as inflammation and ossification (Figure 7D), consistent with the bulk RNA‐seq results (Figure 7A,B). Pseudotime analysis confirmed that VIC groups 1 and 3 have the potential to generate groups 2, 4, or 5 VICs (Figure 7E). These results unraveled a similarity in VIC clustering between mice and humans. Moreover, we detected increased expression of inflammatory cytokines by 2 subgroups of macrophages (Figure S13A,B, Supporting Information). T cells also showed activation in patients with CAVD, although they could not be further divided into subgroups due to the low number of cells sequenced (Figure S13C, Supporting Information). Moreover, analysis of endothelial cells revealed that they could be divided into 3 subgroups, with one subgroup being similar to the mouse *Prox1^+^* subgroup and expressing much higher levels of inflammatory cytokines, though *PROX1* was not expressed in endothelial cells of the human valve (Figure S13D–F, Supporting Information). Importantly, scRNA‐seq analysis also revealed decreased expression of some vitamin D metabolism and response genes, including *CYP27A1*, *FGFR1*, and *TRIM25* in the valves of patients with CAVD (Figure 7F). Overall, these results suggest that human valvular calcification might involve pathologic mechanisms similar to those in mice.

## 3 Discussion

Previous studies suggest that phosphate induces valve calcification by forming Ca‐Pi crystals that damage VECs and cause inflammation, or by directly activating NF‐κB to stimulate BMP expression in valvular cells. Here, we show that high Pi suppresses the expression of vitamin D metabolism genes, especially in VICs, reducing the levels of 1,25(OH)_2_D. This promotes inflammation that involves CD4^−^CD8⁺ T cells, *Prox1⁺* lymphatic endothelial cells, and macrophages. The resulting cytokines, together with Pi ions, induce osteogenic differentiation of two subgroups of *Prrx1⁺Acta2^−^* VICs via activation of ERK signaling, ultimately causing valvular calcification. Importantly, many of these pathological changes were also observed in CAVD patient samples. This study identifies the specific cell types involved in valve inflammation and calcification, as well as the mechanisms governing VIC osteogenic differentiation, thereby advancing our understanding of the pathogenesis of CAVD.

Previous studies have suggested that CAVD is caused by the osteogenic differentiation of VICs or VECs, both of which are highly heterogeneous.^[^ ^3^, ^5^, ^6^, ^12^ ^]^ Two early studies identified 3 (postnatal day 7) and 4 subgroups (postnatal day 30) of VICs in mice.^[^ ^9^ ^]^ In humans, 3 VIC populations have been identified in normal valves, whereas 5 more subgroups have been identified in calcified valves, originating from VICs or VECs.^[^ ^15^ ^]^ Our scRNA‐seq analysis identified 5 subgroups of VICs in mice with no emergence of new cell subpopulations or osteogenic differentiation of VECs in HPD mice. However, only two subpopulations of VICs are expanded in response to HPD, and they express the highest levels of osteogenic and chondrogenic differentiation markers, indicating that they are the primary VICs contributing to valvular calcification. Our scRNA‐seq analysis of valve samples from patients with CAVD also revealed 5 groups of VICs with 2 groups undergoing osteogenic differentiation, similar to HPD mice. The discrepancy in our and others’ cell clustering is likely caused by differences in sequencing depth and cell clustering strategies and warrants further investigation. Moreover, our genetic tracing experiments indicated that *Prrx1* could be used as a genetic marker for all VICs, whereas *Acta2* could be used as a pericyte marker (group 2 VICs) in the valve. The two subgroups of VICs that were actively involved in valvular calcification were *Prrx1^+^Acta2^−^*. These findings help elucidate the cellular basis of valvular calcification in the mouse, and likely in humans.

Inflammation plays a crucial role in the development of CAVD.^[^ ^19^, ^21^ ^]^ Previous studies have suggested that phosphate crystals and phosphate‐generated ROS contribute to cell damage and/or senescence, thereby triggering inflammatory responses.^[^ ^33^ ^]^ In this study, we demonstrated that HPD‐induced valve inflammation was associated with impairments in vitamin D synthesis, especially in the valve. This is supported by our findings that 1,25(OH)_2_D, but not the inactive vitamin D, ameliorated both valvular inflammation and calcification. In particular, high phosphate levels suppressed the expression of vitamin D metabolism genes, especially *Cyp27a1*, in VICs but not in the kidney or liver. Moreover, the valve samples of patients with CAVD also presented reduced expression of vitamin D metabolism and response genes, including *Cyp27a1*. This, together with the recent findings that active vitamin D inhibits T cells and other immune cells via paracrine mechanisms^[^ ^57^ ^]^ suggests that the deficiency of active vitamin D induces the activation of valve CD4^−^CD8^+^ T cells, *Prox1^+^* lymphatic endothelial cells, and macrophages in the valve. Overall, these results imply that valvular calcification is attributable to active vitamin D deficiency‐induced inflammation.

This study also uncovered the molecular mechanisms by which high Pi induces osteogenic differentiation of VICs. Previous studies have suggested that phosphate or inflammation promotes VIC osteogenic/chondrogenic differentiation via NF‐κB signaling in vitro.^[^ ^18^, ^51^ ^]^ Here, we showed that inflammatory cytokines and Pi ions induced VIC osteogenic differentiation cooperatively or synergistically in an ERK‐dependent manner. It is possible that cytokines and Pi ions converge on ERK signaling to promote VIC osteogenic differentiation. Furthermore, this study does not support the role of the BMP, Wnt, or NF‐κB signaling pathway in high Pi‐induced VIC osteogenic differentiation. It is likely that these pathways have redundant functions in promoting VIC osteogenic differentiation. These results highlight the importance of ERK signaling in valve calcification, similar to its role in bone remodeling.^[^ ^58^ ^]^

Previous studies have reported contradictory results regarding the roles of vitamin D in valvular calcification.^[^ ^36^, ^49^ ^]^ In this study, we measured the levels of 1,25(OH)_2_D and detected a greater reduction in the valves than in the serum in HPD mice. These findings, together with our observation that the active but not the inactive form of vitamin D prevents HPD‐induced valvular calcification and inflammation, highlight the importance of local vitamin D metabolism in controlling inflammation and the pathogenesis of valve calcification. This explains why supplementation with vitamin D may not be effective, as shown in two clinical studies.^[^ ^50^, ^59^ ^]^ Due to the uncertainty surrounding the reversal of valve calcification, active vitamin D may be considered for preventing CAVD, although this potential use and the dose require further validation.

This study has several limitations. First, the number of patient samples analyzed in this study is limited due to the difficulty in collecting human cardiac valve specimens. This contributes to the scarcity of spatial single‐cell transcriptomic data from CAVD patients. Second, genetic evidence from VIC‐specific knockout models of vitamin D metabolism genes is currently unavailable. Third, while *Prox1⁺* valvular endothelial cells appear to be involved in valve pathology, our study lacks a detailed mechanistic investigation of their role, partly due to challenges such as the absence of specific genetic tools and the complexity of these endothelial subpopulations.

In summary, this study identified specific VIC subpopulations responsible for valvular calcification induced by hyperphosphatemia, delineated the molecular pathways driving VIC osteogenic differentiation, and highlighted active vitamin D as a potential preventive treatment for CAVD in patients with CKD. Since aging is a major risk factor for CAVD and also leads to defects in vitamin D metabolism,^[^ ^55^, ^56^ ^]^ we propose that impaired vitamin D metabolism may represent a common pathogenic factor in CAVD development.

## 4 Experimental Section

### Mice

Normal mice used in this study were purchased from the Model Animal Research Centre (MARC) of Nanjing University. Male C57BL/6 mice (8 weeks old) were used in the experiments. The knock‐in *Prrx1‐CreERT^2^* and *Vimentin‐CreERT^2^* mouse lines were generated by Shanghai Biomodel Organism Science & Technology Development Co., Ltd. *Rosa26LSL‐tdTomato*, *Acta2‐CreERT^2^*, *Col1a2‐CreERT^2^*, and *Gli1‐CreERT^2^* mice were purchased from the Jackson Laboratory (https://www.jax.org/cn/). All the mice were raised in the specific pathogen‐free Laboratory Animal Centre of Shanghai Jiao Tong University. For valve calcification model generation, mice were fed a high‐phosphorus diet for 4–5 months. The content of phosphorus or calcium in the diet was increased to 2.5% or 5% based on the AIC93G diet, and other components remain unchanged. The study was approved by the ethics committee of Shanghai Jiao Tong University (A2024014 and 2024132‐001). The numbers of mice used for each experiment were designated in the figure legends, with no data excluded.

### Vitamin D and Drug Administration

The calcitriol powder (S1466, Selleck) was dissolved in 100% ethanol and subsequently diluted in 5% ethanol to appropriate concentrations, and mice received calcitriol (100 ng kg^−1^ body weight) via gastric gavage once every other day for 4–5 months, as previously reported.^[^ ^60^ ^]^ This is a low dose compared to other studies.^[^ ^61^, ^62^ ^]^ Vitamin D3 (S4063, Selleck) was dissolved in dimethylformamide and subsequently diluted in corn oil. A range of 50–625 µg kg^−1^ body weight of vitamin D3 has been used in previous studies.^[^ ^63^, ^64^, ^65^ ^]^ At 625 µg kg^−1^ (via gastric gavage once every other day), the mice became weak; still, it did not rescue the valve calcification phenotype. Lower doses were tested and found that doses >62.5 µg kg^−1^ could cause the loss of body weight. Vitamin D at the dose of 62.5 µg kg^−1^ was thus used in this study.

U0126 (S1102, Selleck) was dissolved in 0.5% sodium carboxymethyl cellulose (CMC‐Na) and administered by oral gavage to mice at 20 mg kg^−1^ every other day. LDN‐193189 2HCl (S7507, Selleck) was dissolved in 0.9% saline and administered by i.p. to mice at 2 mg kg^−1^ every other day.

### X‐Ray Imaging and Ultrasound

The heart images were taken using a Cabinet X‐Ray system (LX‐60, Faxitron Bioptics) with standardized settings (45 Kv for 7 s).

Ultrasound scans were performed using a US/PA Imaging system (VEVO LAZR‐X, Fujifilm Visual Sonics). Mice (N = 3) were placed on the platform, and the mouse's mouth and nose were inserted into an anaesthesia mask to maintain anaesthesia with isoflurane at a concentration of 1–2% and a flow rate of 0.6–1 L min^−1^. Heart function was measured with the “Cardiac Package”. The severity of calcification is determined by transaortic valve pressure and transaortic valve velocity.

### Genetic Lineage Tracing

For genetic lineage tracing, TAM (Sigma, T5648) dissolved in corn oil was injected (i.p.) into adult *Tomato^Prrx1^* or *Tomato^Acta2^* male mice at 120 mg kg^−1^ body weight daily for 3 days, and collected at different time points.

### Organ Collection and Histology

Mice were anesthetized using isoflurane, and then the hearts were excised following perfusion with PBS. The hearts were fixed in 4% PBS‐buffered paraformaldehyde overnight at 4 °C. The samples were dehydrated in alcohol, cleared with xylene, and embedded in paraffin. Four‐µm‐thick sections were cut using a microtome (Leica Microsystems Nussle GmbH). Hematoxylin and eosin (H/E) or Von Kossa staining was carried out. Stained slides were photographed under a light microscope (Olympus Microsystems).

### Immunofluorescence and Immunohistochemical Staining

Heart sections were deparaffinized using xylene and rehydrated with gradient ethanol. They were further permeabilized with 0.1% Triton X‐100 for 30 min at room temperature. Subsequently, antigen retrieval was done in 99 °C citric acid buffer for 20 min. The sections were blocked with 5% bovine serum for 60 min and incubated with primary antibodies (1:100) at 4 °C overnight, and with secondary antibodies for 60 min at 37 °C. The primary antibodies used in this study were: αSMA (ab5694, Abcam); RUNX2 (12556S, Cell Signaling Technology); Osx (ab22552, Abcam); Aggrecan (AB1031, EMD Millipore); Sox9 (AB5535, EMD Millipore); p‐p44/42 MAPK (9101S, Cell Signaling Technology); p‐NF‐κB (p65, RelA) (3033S, Cell Signaling Technology); p‐Stat3 (9145S, Cell Signaling Technology); CD31 (ab56299, Abcam); CD45 (ab10558, Abcam); Vimentin (ab92547, Abcam); F4/80 (ab6640, Abcam); CYP27B1 (F3415, Sellcek); VDR (sc‐1008, Santa Cruz); Lyve‐1 (103‐PA50AG, ReliaTech). The secondary antibodies were goat anti‐rabbit or goat anti‐rat conjugated to Alexa Fluor 488 (Invitrogen). The slides were mounted with anti‐fade mounting medium with DAPI (Sigma‐Aldrich). Images were taken under a confocal microscope (Leica TCS SP8).

### VICs Culture

Aortic valves were obtained from adult mice. First, mouse valve tissues were isolated under a stereoscope, and the samples were cut into small pieces, digested with 2 mg mL^−1^ collagenase type II (Invitrogen) at 37 °C with 5% CO_2_ for 45 min. Digestion was terminated by adding 3 times the complete medium. Subsequently, cells were sieved through a 70 µm filter, centrifuged at 1300 rpm for 5 min, resuspended, and seeded in αMEM medium containing 10% FBS.

### In Vitro Osteogenic Differentiation Assays

To determine the effects of high phosphate and LPS on VIC osteogenic differentiation, primary mouse VICs were cultured with 2 mm Pi, 1 µg mL^−1^ LPS, or 2 mm Pi in combination with 1 µg mL^−1^ LPS for 6 days at 37 °C with 5% CO_2_. To determine the effects of ERK or NF‐κB signaling on VIC osteogenic differentiation, VICs were preincubated with QNZ (500 ng mL^−1^, Selleck, EVP493) or U0126 (10 µm, Targetmol, T6223) for 2 h, and then treated with Pi, LPS, or Pi plus LPS at 37 °C with 5% CO_2_. To determine the effects of calcitriol on VIC osteogenic differentiation, VICs were incubated with calcitriol for 2 h and then treated with Pi, LPS, or both. To determine the effects of Wnt signaling on VIC osteogenic differentiation, VICs were preincubated with IWR‐1 (5, 10, or 20 µm, MedChemExpress, HY‐12238) for 2 h, and then treated with Pi, LPS, or both at 37 °C with 5% CO_2_. Osteogenic differentiation and calcification were detected with ALP and Alizarin Red S staining, respectively. qPCR was conducted to determine the expression of osteoblast marker genes such as *Alp*, *Runx2*, and *Osteocalcin* (*Ocn*).

### Flow Cytometry

To analyze immune cells, digested valvular cells were stained with the following antibodies: CD45‐PE594, CD3E‐PE, CD4‐PE‐Cy7, CD8A‐APC, F4/80‐FITC, LY6G‐BV711. All staining was performed for 1 h on ice. Dead cells were identified and gated out of sorts by LIVE/DEAD fixable dead cell stain kits. Single‐staining tubes were used to set compensation. Flow cytometry analysis was performed on BD Aria II with software FACSDiva (v6.1.3). The FACS data were analyzed with Flowjo (v10.5.3), and all figures were displayed in the Biex mode.

### Western Blot

The cells were scraped off the culture dish on ice and boiled. The protein concentrations of the samples were determined using a BCA assay. For Western blot analysis, equal amounts of proteins were loaded onto 10% SDS‐PAGE gels and transferred to polyvinylidene fluoride (PVDF) membranes (Sigma‐Aldrich). Membranes were blocked with 5% non‐fat milk for 1 h at room temperature. The membranes were then incubated overnight at 4 °C with primary antibodies (1:1000) against Actin (Sc‐47778, Santa Cruz Biotechnology); p‐NF‐κB (p65, RelA) (3033S, Cell Signaling Technology); NF‐κB (p65, RelA) (8242S, Cell Signaling Technology); p‐p44/42 MAPK (9101S, Cell Signaling Technology); p44/42 MAPK (9102, Cell Signaling Technology). The membranes were then incubated with appropriate horseradish peroxidase (HRP)‐conjugated secondary antibodies for 1 h at room temperature. Immunoreactive bands were visualized using enhanced chemiluminescence (ECL) substrate (Tanon 5200, Shanghai).

For Western blot analysis of nuclear and cytoplasmic p‐ERKs, the nucleus and cytoplasm were prepared using a nuclear and cytoplasmic protein extraction kit (P0028, Beyotime).

### RNA Isolation and Quantitative PCR

For RNA extraction, valve tissues or cultured VICs were collected and homogenized in TRIzol reagent (Invitrogen) using a tissue homogenizer (JXFSTPRP‐CL, Shanghai) at 65 Hz for 10 min. The homogenate was then subjected to phase separation by adding chloroform (Sigma‐Aldrich), followed by centrifugation at 12000 g for 15 min at 4 °C. The upper aqueous phase was carefully transferred to a new tube, and RNA was precipitated by adding isopropanol. The samples were then centrifuged at 12 000 g for 10 min at 4 °C. The RNA pellet was washed with 75% ethanol, air‐dried, and resuspended in RNase‐free water. RNA quality and concentration were assessed using a NanoDrop 2000 spectrophotometer (Thermo Fisher Scientific).

For qPCR analysis, 1 µg of extracted RNA was reverse transcribed using the High‐Capacity cDNA Reverse Transcription Kit (Thermo Fisher Scientific). The resulting cDNA was diluted to 10 ng µL^−1^ and used for qPCR with SYBR Green Master Mix (Invitrogen) in a final volume of 20 µL. The amplification was performed using Light Cycle 480 under the following conditions: 95 °C for 10 min, followed by 40 cycles of 95 °C for 15 s, and 60 °C for 1 min. Primer sequences were designed using NCBI Primer‐BLAST and validated for efficiency. The relative expression levels were calculated using the 2^(‐ΔΔCt) method, with GAPDH as the reference gene. The primer sequences are:

**Table:** 

Gene	Forward Primer (5′ to 3′)	Reverse Primer (5′ to 3′)
*Gapdh*	CATCACTGCCACCCAGAAGACTG	ATGCCAGTGAGCTTCCCGTTCAG
*Alp*	ATGCCCTGAAACTCCAAA	AGACGCCCATACCATCTC
*Runx2*	GACTGTGGTTACCGTCATGGC	ACTTGGTTTTTCATAACAGCGGA
*Ocn*	CAGACAAGTCCCACACAG	GCAGAGTGAGCAGAAAGA
*Wnt3a*	AACTGCACCACCGTCAGCAACA	AGCGTGTCACTGCGAAAGCTAC
*Wnt5a*	GGAACGAATCCACGCTAAGGGT	AGCACGTCTTGAGGCTACAGGA
*Bmp2*	GGGACCCGCTGTCTTCTAGT	TCAACTCAAATTCGCTGAGGAC
*Bmp4*	TTCCTGGTAACCGAATGCTGA	CCTGAATCTCGGCGACTTTTT
*Bmp5*	TTACTTAGGGGTATTGTGGGCT	CCGTCTCTCATGGTTCCGTAG
*Tnfa*	CCCTCACACTCAGATCATCTTCT	GCTACGACGTGGGCTACAG
*Il1b*	GCAACTGTTCCTGAACTCAACT	ATCTTTTGGGGTCCGTCAACT
*Il6*	GTTGCCTTCTTGGGACTGATG	GACTCTGGCTTTGTCTTTCTTGTT
*Cxcl12*	CCAACCACCAGGCTACAGG	GCGTCACACTCAAGCTCTG
*Il17a*	CGCAATGAAGACCCTGATAGAT	CTCTTGCTGGATGAGAACAGAA
*Cyp27a1*	CGGGGACCGGAACGCTA	TTGGTCTTGTTCAGCACCTGGA
*Cyp27b1*	TCCCAGACAGAGACATCCGT	GTAGCTCAAGCTCTGCCAAG
*Cyp24a1*	CAAGTGCAACAGAGACTTCTCC	TTGTCTTCGCTAGAGCCCAG
*Cyp2r1*	CAACAGGAGACCTTCTTGGGA	TTGCTGTCCAGAAATCGCTC
*Vdr*	CCGGTTCCATCATGTCCAGT	GCTATTCTCCAAGGCCCACA
*Fgfr1*	CCCAGGGCTGAGCTTGTT	ACGGAGCCTTGTTACCAACC

### VD, 25(OH)D, 1,25(OH)_2_D, Pi and Calcium Measurement

Serum Pi and calcium were measured using the Phosphate Assay Kit and Calcium Assay Kit, respectively (BioAssay Systems). Serum VD and 25(OH)D were quantified by HPLC‐MS at the Instrumental Analysis Center, Shanghai Jiao Tong University. Serum and valvular tissue 1,25(OH)_2_D were measured using Universal 1,25‐dihydroxyvitamin D3(DVD/DHVD3) ELISA Kit (NBP2‐82432, NOVUS Biologicals). For tissue homogenates, mince the tissues into small pieces and thoroughly rinse them with ice‐cold PBS (0.01 m, pH 7.4) to remove excess blood. The tissue pieces were weighed, then homogenized in PBS at a ratio of 1 g tissue to 9 mL PBS using a glass homogenizer on ice. To further disrupt the cells, the suspension was sonicated with an ultrasonic cell disruptor and subjected to freeze‐thaw cycles. The homogenates were centrifuged at 5000 × g for 5 min to collect the supernatant. For serum preparation, blood samples were allowed to clot at room temperature for 2 h or at 2–8 °C overnight. A total of 1000 × g was centrifuged for 15 min at 2–8 °C, and the supernatant was collected for assays.

### Ex Vivo Valve Vitamin D Metabolism Assay

A previously reported protocol was used.^[^ ^66^, ^67^ ^]^ Briefly, C57BL/6 mice were sacrificed in a CO_2_ chamber, and the aortic valve was collected in αMEM supplemented with 15% FBS and 100 µm vitamin D and incubated for 72 h at 37 °C and 5% CO_2_. For the ELISA assay, the tissue was processed according to the manufacturer's instructions of the Universal 1,25‐dihydroxyvitamin D3(DVD/DHVD3) ELISA Kit (NBP2‐82432, NOVUS Biologicals).

### Bulk RNA‐Seq

The heart valves (3 control and 3 HPD mice) were isolated immediately after euthanization and washed with cold PBS, centrifuged at 300 *g* for 3 min to remove excess liquid, harvested using TRIzol reagent (Invitrogen, CA), and finally delivered on dry ice to BGI Wuhan Research Centre for RNA sequencing. All differentially expressed genes (DEGs) were submitted to the online tool Dr. TOM software for gene ontology and KEGG pathway enrichment analysis. For the DEGs, *p* < 0.05 was accepted as significant.

### Single Cell RNA Sequencing

The valves of control and HPD mice were isolated immediately after euthanization and washed with cold PBS, followed by mechanical dissociation using scissors. The dissociated samples were digested in αMEM with collagenase type II, 2 mg mL^−1^ (Invitrogen, CA) for 45 min to prepare a single‐cell suspension, then incubated with Calcein‐AM (40719ES50, Yeasen) for 10 min at 37 °C for FACS sorting using BioRad S3e. Isolated live cells were resuspended in PBS containing 0.1% BSA. The single‐cell suspension was loaded into chromium microfluidic chips with 3′ v3 chemistry and barcoded using a 10x Chromium Controller (10x Genomics). Briefly, 9000 cells were loaded into each reaction, and the RNA transcripts from individual cells were uniquely barcoded and reverse‐transcribed. cDNA molecules were pre‐amplified, fragmented, end‐repaired, and ligated with adaptors as per the manufacturer's protocol in order to generate a single multiplexed library for sequencing on an Illumina NovaSeq 6000 (Illumina).

### Analysis of scRNA‐Seq Data

The raw sequencing data were processed with Cell Ranger (v3.0.1). Cell Ranger counts were then used to map reads to the mouse reference genome. The generated expression matrices (gene‐cell expression matrices) were used for subsequent analysis in R version 4.2.2 using Seurat version 4.3.0.1. “Cells” that fit any of the following criteria were excluded: <200 expressed genes, >20% UMIs mapped to mitochondria. Filtered cells were used for downstream graph‐based clustering and t‐SNE visualization. Violin plots, feature plots, and heatmaps were generated using “Seurat” implemented functions. For cell trajectory analysis, we used “Monocle2” package. To draw the correlation heatmap of 5 VIC clusters, average gene expression data of the total cells in each cluster were calculated, and the top 200 expressed genes were selected. Cellular communication was analyzed using the “CellChat” package. For *Cyp27a1‐Vdr* interaction analysis, the function “updateCellChatDB” was used to update CellChatDB by integrating *Cyp27a1‐Vdr* pairs into the ligand‐receptor interaction database; a connecting line was drawn if gene expression levels exceeded +25%.

### Statistical Analysis

Data were expressed as mean ± standard deviation (SD). Statistical significance between groups was determined using one‐way ANOVA and two‐way ANOVA for multiple comparisons or a unpaired two‐tailed Student's *t*‐test for correlation data; *p* < 0.05 was considered statistically significant. Correlation coefficients (R^2^) were calculated as Pearson's correlation coefficient. In each analysis, the exact value of n was reported for every experimental group. Prism 9.0 and R were used for statistical analysis.

### Ethics

Animal experiments were carried out in accordance with recommendations in the National Research Council Guide for Care and Use of Laboratory Animals and in compliance with protocols approved by the Institutional Animal Care and Use Committee of Shanghai, China (A2024014 and 2024132‐001).

## Conflict of Interest

The authors declare no conflict of interest.

## Author Contributions

R.Y., C.H., Y.X., and S.Q. contributed equally to this work. H.L., L.C., Z.Z., and B. L. conceived and designed experiments. R.Y., C.H., Y.X., S.Q., and J.H. performed the mouse experiments. C.H., Z.W., and J.H. performed cell‐based experiments. R.Y. and S.Z. performed the bioinformatics analysis. R.Y., S.Z., and Z.W. performed flow cytometry experiments. R.Y., C.H., H.L., Y.X., L.C., Z.Z., and B.L. wrote the manuscript with contributions from all other authors. H.L., L.C., and B.L. oversaw the project. All authors read and approved the manuscript.

## Supporting information



Supporting Information

## Data Availability

The data that support the findings of this study are available on request from the corresponding author. The data are not publicly available due to privacy or ethical restrictions.
