# Supplementary material for: The Pathogenic Roles of Local Vitamin D Metabolism Defect in Valve Inflammation and Calcification
Source: Adv Sci (Weinh). 2025 Oct 13;12(48):e01250. doi: 10.1002/advs.202501250 (PMC12752593; doi:10.1002/advs.202501250)
Supplement: Supplementary file 1 — Supporting Information [file ADVS-12-e01250-s001.docx]

**Figure S1**

**Figure S1. High-calcium diet did not induce valvular calcification.**

A. X-ray imaging revealed that high calcium (5%) did not cause calcification in the valve in adult male mice.

B. H/E and Von Kossa staining revealed normal valve structure with no mineralization nodules in high-calcium mice. Scale bars=25 μm.

**Figure S2**

**Figure S2. Enriched expression of inflammation, chemokine and myofibroblast related genes in HPD mice.**

A. Heatmaps of chemokine, chemotaxis, myofibroblast, osteoclast differentiation, and neutrophil activation related genes.

B. qPCR results showing enriched expression of *Wnt* and *BMP* genes in the valves of HPD mice. N=3 mice, with triplicate each.

C. Representative staining results for CD45^+^ immune cells 2 months after HPD, before calcification occurred. Scale bars=25 μm.

D. Representative IHC staining results showing an increase in p-RelA and p-Stat3 in the valve of HPD mice. Scale bars=25 μm.

Unpaired two-tailed Student’s t test were applied to evaluate the correlation data in (B), p<0.05 was considered as statistically significant.

**Figure S3**

**Figure S3. Comparison of each of the VIC groups between HPD and control mice.**

A. GO analyses of 5 VICs.

B. Expression of osteoblast, chondrocyte and myofibroblast genes in the 3 VIC groups of HPD and control mice.

**
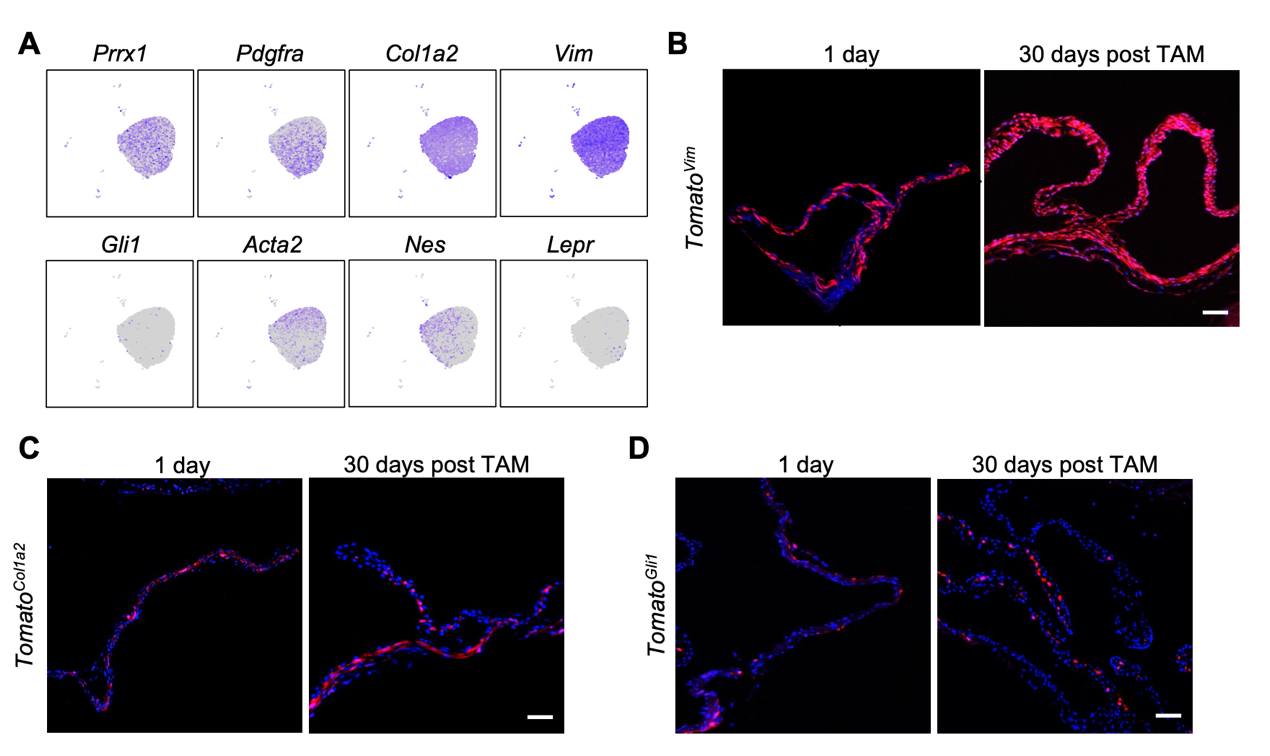
Figure S4**

**Figure S4. Genetic tracing of Vimentin, Col1a2, or Gli1 lineage cells in the valve.**

A. tSNE results showing the expression of mesenchymal/stromal markers in various VIC groups.

B. Tracing results of *Tomato^Vim^* mice. The mice were euthanized on Day 1 or 30 after TAM administration. The valve sections were coimmunostained for Tomato and DAPI. Scale bars=25 μm.

C. Tracing results of *Tomato^Col1a2^* mice. The mice were euthanized on Day 1 or 30 after TAM administration. The valve sections were coimmunostained for Tomato and DAPI. Scale bars=25 μm.

D. Tracing results of *Tomato^Gli1^* mice. The mice were euthanized on Day 1 or 30 after TAM administration. The valve sections were coimmunostained for Tomato and DAPI. Scale bars=25 μm.

**Figure S5**

**Figure S5. Analysis of immune cells in the valve and the peripheral blood.**

A. GO analyses of valvular macrophage subgroup 1 of HPD mice against that of control mice.

B. GO analyses of valvular macrophage subgroup 2 of HPD mice against that of control mice.

C. Gating strategies for immune cell analysis with flow cytometry.

D. Flow cytometry analysis of valve immune cells of HPD and control mice. N=3.

E. Flow cytometry analysis of immune cells in the peripheral blood of HPD and control mice. N=3.

**Figure S6**

**Figure S6. scRNA-seq analysis of valvular endothelial cells.**

A. Clustering of the valvular endothelial cells.

B. Markers used in the clustering of endothelial cells.

C. Representative immunostaining results for Lyve-1 in the valves of HPD and Ctrl mice. Scale bars= 25 μm.

D. GO analyses of endothelial cell subpopulations.

E. Heatmap of cytokines and chemokines expressed in *Prox1^+^* endothelial cells.

**Figure S7**

**
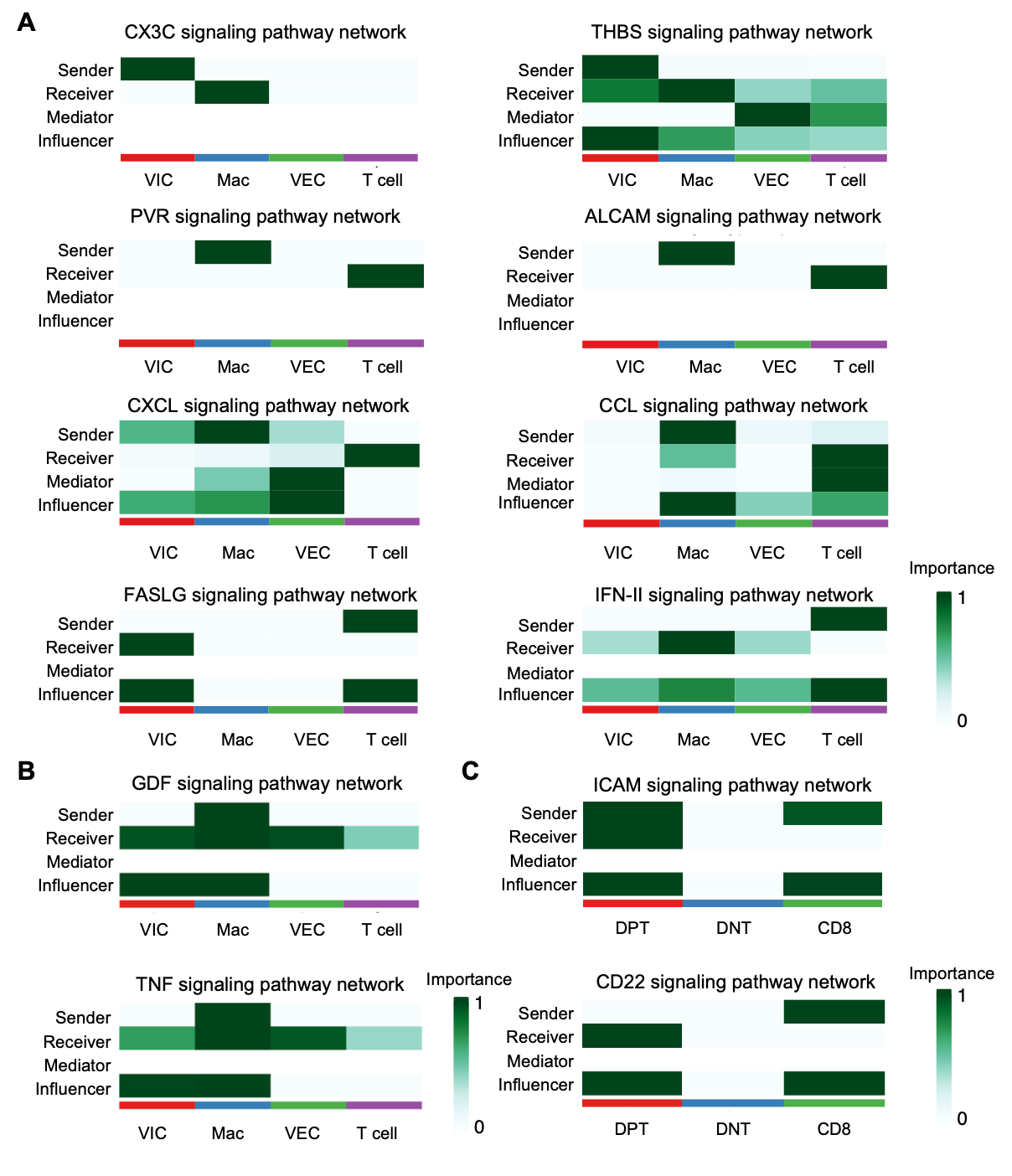
Figure S7. Ligand (inflammatory signals) -receptor interaction analysis of valvular cells.**

A. Immune or inflammation related pathways including CX3C, THBS, PVR, ALCAM, CXCL, CCL, FASLG, and IFN-II were analyzed.

B. Analysis of ligand-receptor communication networks between macrophages and VICs.

C. Analysis of ligand-receptor communication networks between CD8^+^ T cells and other T cells.

**Figure S8**

**Figure S8. Calcitriol but not vitamin D suppressed HPD-induced inflammation.**

A. HPD mice were treated with low doses of calcitriol (20 ng/kg and 50 ng/kg) for 4 months. Representative H/E and Von Kossa staining showed that while 20 ng/kg had no effect, the 50 ng/kg dose partially reversed HPD-induced calcification. Scale bars= 25 μm.

B. X-ray imaging revealed that vitamin D did not affect HPD-induced valvular calcification in adult male mice. The control images of HPD mice were shown in Figure 6A.

C. H/E and Von Kossa staining results of vitamin D-treated HPD mice. The control images of HPD mice were shown in Fig. 6B. Scale bars=25 μm.

D. HPLC-MS assay showed that HPD increased serum levels of Vitamin D and 25(OH)D. N=3.

E. qPCR results showed that calcitriol suppressed elevated expression of *BMP* and *Wnt* genes in the valve of HPD mice. N=3 mice, with triplicate each.

F. tSNE analysis of valvular cells in calcitriol-treated mice on HPD. Right panel: the percentages of various cell groups.

G. tSNE analysis of endothelial cells in calcitriol-treated mice on HPD. Right panel: the percentages of various cell groups.

H. GO analysis showed that the expression of inflammation-related pathway genes in *Prox1^+^* cells was suppressed by calcitriol.

I. Heatmap showed that the expression of inflammatory cytokine of *Prox1^+^* cells was suppressed by calcitriol.

Two-way ANOVA (or mixed model) multiple comparisons were applied to evaluate the correlation data in (E), p<0.05 was considered as statistically significant.

**Figure S9**

**
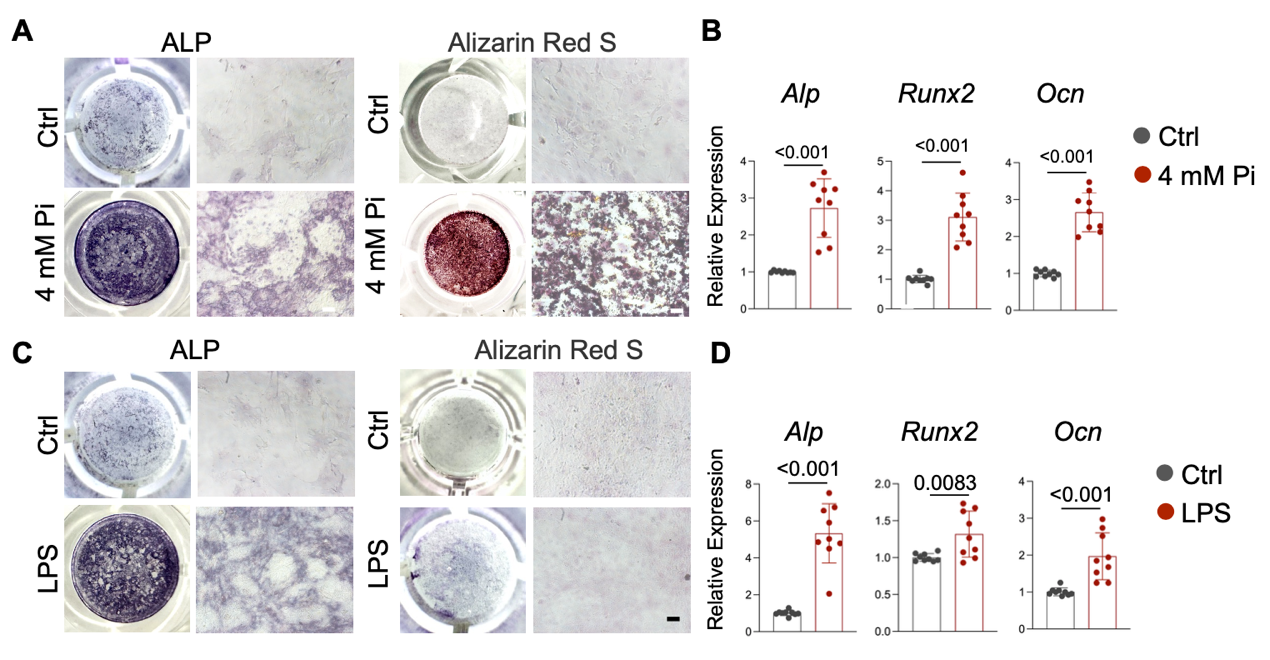
Figure S9. The effect of Pi or LPS on VIC osteogenic differentiation.**

A, B. Pi (4 mM) induced VIC osteogenic differentiation, which was verified by staining for ALP or Alizarin red (A) and qPCR analysis of osteoblast-specific genes (B). Scale bars=100 μm.

C, D. LPS induced VIC osteogenic differentiation, which was verified by staining for ALP or Alizarin red (C) and qPCR analysis of osteoblast-specific genes (D). N=3, with triplicate each. Scale bars=100 μm.

Unpaired two-tailed Student’s t test were applied to evaluate the correlation data in (B and D), p<0.05 was considered as statistically significant.

**Figure S10**

**Figure S10. The effect of NF-κB or Wnt inhibitor or calcitriol on VIC osteogenic differentiation.**

A. KEGG and GO analyses of VICs (combined all 5 subpopulations) from scRNA-seq data of Ctrl and HPD mice.

B. LPS but not Pi (4 mM) activated NF-κB in VICs. Cells were collected 4 hours after treatment.

C, D. Blockade of NF-κB with QNZ (500 ng/mL) did not affect Pi-induced VIC osteogenic differentiation, which was verified by staining for Alizarin red (C) and qPCR analysis of osteoblast-specific genes (D). N=3, with triplicate each. Scale bars=100 μm.

E, F. Blockade of [β-catenin](https://www.medchemexpress.cn/Targets/(beta)-catenin.html) activation with IWR-1 (20 μM) did not affect Pi-induced VIC osteogenic differentiation, which was verified by staining for Alizarin red (E) and qPCR analysis of osteoblast-specific genes (F). N=3.

G. Western blot results showed that Pi (4 mM) and LPS did not alter the level of p-ERK in VICs. Cells were collected 12 hours after treatment.

H. Pi (4 mM) and LPS induced nuclear localization of activated ERKs in VICs. The cells were collected 12 hours after treatment. Scale bars=25 μm.

I, J. Calcitriol (5 μM) did not affect Pi and LPS-induced VIC osteogenic differentiation, which was verified by staining for Alizarin red (I) and qPCR analysis of osteoblast-specific genes (J). N=3, with triplicate each. Scale bars=100 μm.

K. qPCR analysis shows high doses of calcitriol did not affect the phosphate (Pi) and LPS-induced expression *Runx2*. N=3.

Two-way ANOVA (or mixed model) multiple comparisons were applied to evaluate the correlation data in (D, F and J), p<0.05 was considered as statistically significant. Unpaired two-tailed Student’s t test were applied to evaluate the correlation data in (K), p<0.05 was considered as statistically significant.

**Figure S11**

**Figure S11. RNA-seq analysis of CAVD patient transcriptomes.**

A. KEGG and GO analyses were performed on the bulk RNA-seq data from GSE199718 (N=7 for control, N=5 for patients).

B. TPM values of vitamin D metabolism and response genes were analyzed in valve samples from patients with CAVD and controls in dataset GSE199718.

**Figure S12**

**Figure S12. scRNA-seq analysis of VICs of CAVD patients.**

A. tSNE analyses of valvular cells of patients with CAVD and control. Right panel: percentages of various populations.

B. Markers used for clustering various cell populations.

C. GO analyses of various VIC groups of human.

**Figure S13**

**Figure S13. RNA-seq analysis of immune and endothelial cells of CAVD patients.**

A. tSNE analysis of valvular macrophages of patients with CAVD.

B. GO analysis of valvular macrophages of patients with CAVD.

C. GO analysis of valvular T cells of patients with CAVD.

D. tSNE analysis of valvular VECs of patients with CAVD.

E. Markers used for VEC clustering.

F. GO analysis showed enriched expression of inflammation-related genes in the subgroup of VECs of the patients with CAVD.
